# Bundling gold nanorods with RCA-produced DNA tape into an intelligently reconfigurable nanocluster bomb for multimodal precision cancer therapy

**DOI:** 10.1016/j.mtbio.2025.101718

**Published:** 2025-03-28

**Authors:** Qian Gao, Weijun Wang, Shujuan Sun, Ya Yang, Kaili Mao, Yuxi Yang, Zai-Sheng Wu

**Affiliations:** a Cancer Metastasis Alert and Prevention Center, Fujian Provincial Key Laboratory of Cancer Metastasis Chemoprevention and Chemotherapy, State Key Laboratory of Photocatalysis on Energy and Environment, College of Chemistry, Fuzhou University, Fuzhou, 305108, China; b Key Laboratory of Laboratory Medicine, Ministry of Education of China, and Zhejiang Provincial Key Laboratory of Medical Genetics, School of Laboratory Medicine and Life Sciences, Wenzhou Medical University, Wenzhou, 325035, China; c College of Chemistry and Food Science, Nanchang Normal University, Nanchang, 330032, China

**Keywords:** RCA-Based adhesive tape, Bundling gold nanorods into a reconfigurable nanocluster, Explosive cluster bomb for precise combination chemotherapy, Multimodal synergetic cancer therapy

## Abstract

Via proposing an innovative assembly technique, we bundle cell-targeting aptamer-modified gold nanorods (AuNRs) with RCA product (RCA-p) tape into a reconfigurable nanocluster (ARGN) bomb for multimodal precision cancer therapy. Because each ARGN has 10 individual AuNRs, the short time of laser irradiation can make the temperature increase to 75 °C much higher than the lethal temperature of tumor cells, enabling the efficient photothermal therapy (PTT). Moreover, both siRNA-Plk1 (2820 per ARGN) and chemotherapeutic agents (15860 per ARGN) can be loaded into two specifically-designed containers in the internal cavity. Because the glomeroplasmatic structure enhances the resistance to enzymatic degradation, ARGN bomb can protect siRNAs from the digestion and avoid Dox leakage during *in vivo* circulation. Moreover, the spontaneous structural reorganization allows aptamers in the interior cavity move outward to the exterior surface, which magically offers the compensation of degraded aptamers and impair persistent *in vivo* cell targeting ability. The external stimuli (laser irradiation) promotes the release of chemotherapeutic agents and initiates the PTT/chemotherapy outcome, while endogenous stimuli (intracellular biomarkers) causes almost 100 % release of siRNA-Plk1 species and induces RNA interference therapy, completely inhibiting tumor growth without detectable off-target toxicity.

## Highlights

• Aptamer-modified gold nanorods are bundled into a reconfigurable nanocluster (ARGN) bomb with RCA tape for cancer therapy.
• ARGN bomb displays a persistent tumor-targeting ability, ensuring the sufficient accumulation of drugs in tumor site.
• RNAi-/Chemo-/PTT multimodal synergistic therapy based on ARGN bomb almost completely inhibits tumor growth *in vivo*.
• DS-ARGN device has no detectable side effects and becomes an exemplary system of multimodal precision cancer therapy.

## Graphical abstract

**Figure:**
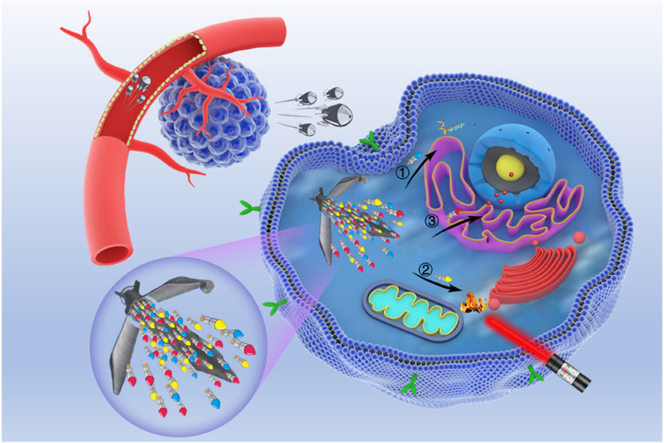

## 1 Introduction

Malignant tumor is a serious threat to human life and health and becomes a major public health problem worldwide. Despite their direct primary resection and high cytotoxicity to tumor cells, traditional treatment methods in clinic, such as resection surgery, chemotherapy and radiotherapy, often suffer the recurrence because of incomplete resection during surgery [1,2] or easily lead to toxic side effects due to the off-target delivery of anticancer agents [3,4]. For example, Doxorubicin (Dox) is a chemical drug widely used for the treatment of a variety of malignant tumors and exhibits a good anti-cancer effect [5]. But, due to the lack of targeting ability, the clinic application of Dox is greatly limited by serious dose-dependent cardiotoxicity [6]. In order to meet the urgent demands for efficient cancer therapy, a variety of new strategies have been extensively explored, including gene therapy, photodynamic therapy (PDT), photothermal therapy (PTT) and immunotherapy. Of them, gene therapy based on small interfering RNAs (siRNA) can target and eliminate the expression of nearly any oncogene in the body or exert the direct knockout of the disease-causing genes of invading viruses, becoming a very broad and promising anti-cancer therapeutic strategy [[7], [8], [9]]. However, although siRNA therapy has been successful in clinical trials to suppress the gene expression of non-tumor diseases, several formidable challenges, including low therapeutic effect, vulnerable degradability and potential safety concerns stemming from off-target effects, put a brake on its rapid clinical adoption [10]. Native natural siRNA has a short circulation time and usually last only a few minutes in the blood [11], making it difficult to reach cancer cells after systematical administration in tumor-bearing mice. The off-target release of siRNA would lead to the accumulation in non-target tissues, causing the systemic toxicity [12,13]. Moreover, negatively charged siRNA is difficult to cross cell membrane by itself and taken up by diseased cells owing to electrostatic repulsion, leading to inadequate and inefficient cellular association and disabling the entry of siRNAs in intact form into the cytosol for gene silencing [9,14]. Therefore, to promote the transition of siRNAs from a research setting to clinical practice of tumor treatment, the development of versatile cancer-targeting nano-scale carriers is urgently needed [5]. For this purpose, several crucial technical innovations are necessary: the *in vivo* stability of nano-carrier should be enhanced to protect siRNA from degradation, the biodistribution upon systemic administration should be directed to cancerous tissues, the uptake efficiency of siRNA by cancer cells should be improved, and the specific stimuli-responsive release of siRNA at the treatment site should be achieved. Meanwhile, PTT therapy is a minimally invasive treatment that converts light energy into heat energy under the irradiation of NIR laser [15,16]. Various nanomaterials with photothermal effect have been widely explored and find the great application in bioimaging and cancer treatment. Due to high NIR absorption efficiency, desirable photothermal conversion capability, ease of synthesis and modification, a myriad of functional biomolecules available for surface functionalization and good biocompatibility, gold nanorod (AuNR) is finding the increasing applications for the imaging and elimination of different tumors [17,18]. Although significant progress has been made in the treatment of cancers, single drug-based monotherapy remains a formidable challenge in eradicating solid tumors mainly due to the inherent heterogeneity and complexity of tumor [[19], [20], [21]], while the combination treatment of multiple therapeutic agents is a more commonly-adopted therapeutic regime in clinic practice due to the synergistic enhancement in the efficacy [22,23]. In order to meet these challenges and improve the therapeutic outcomes, a multimodal combination-based cancer treatment, especially the construction of intelligent multifunctional anticancer biomaterials for multimodal therapy, has become a promising platform for precision cancer therapy [[24], [25], [26], [27], [28], [29]].

As an endogenous genetic material, DNA has the advantages of programmability, biocompatibility and predictability, and is widely used as building block for the construction of DNA nanostructures [[30], [31], [32]]. Moreover, a variety of DNA nanodevices have been demonstrated to play an important role in the fields of biosensing, bioimaging, targeted delivery of drugs and controlled release [[33], [34], [35]]. Rolling circle amplification (RCA) is a polymerase-mediated isothermal nucleic acid amplification technique that can efficiently generate a long tandemly repetitive DNA strand. By deliberately designing a specific template, a sufficiently long single-stranded multifunctional RCA product (RCA-p) can be synthesized through RCA reaction and used as the scaffold for the assembly of DNA nanostructures having the wide range of applications in biomedicine and bioengineering, such as drug delivery [36,37], anticancer immunotherapy [38], combination cancer therapy [39] and precise organization of periodic proteins [40]. Nevertheless, due to the lack of technical guideline for the RCA-p-mediated organization of photothermal transduction agents in a highly ordered fashion, there has been no any report on the assembly of multifunctional photothermal AuNR aggregates capable of performing the structural reconfiguration upon the environmental stimuli with the help of RCA-p strand so far.

Moreover, it is worth mentioning that functional nucleic acids, such as aptamers, have attracted extensive attention in the specific functionalization of DNA nanodevices [[41], [42], [43]] for improving the effectiveness of DNA nanodevices-based targeted therapy against solid tumors. Aptamers are single-stranded DNA or RNA obtained through the Systematic Evolution of Ligands by Exponential Enrichment (SELEX) technology. They have a high affinity for a variety of specific targets, including metal ions, peptides, proteins, viruses, whole cells and even target species within living organisms [[44], [45], [46]]. Modification of a drug carrier, especially a non-nucleotide acid nano-object, with aptamers makes the surfaced-confined functional strands to hold an enhanced resistance to nuclease degradation for ensuring the long circulation time and promoting tumor cell internalization remains a major challenge.

In this contribution, we demonstrate a multimodal cancer treatment platform, reconfigurable DS-ARGN cluster bomb, where gold nanorods are bundled together with RCA-p adhesive tape and densely loaded with Dox and siRNA of PLK1 gene (siRNA-Plk1) to simultaneously execute photothermal therapy, gene therapy and chemotherapy for achieving synergistic precision treatment effects. Specifically, an oligonucleotide sequence (Apt-anchor) containing dual-AS1411 aptamer is attached onto the surface of AuNR via thiol-gold chemistry, generating A-AuNR. Then, a specifically-synthesized RCA-p strand complementary to Apt-anchor is used as the adhesive tape to bundle several A-AuNRs together, forming ARGN carrier. Moreover, siRNA-Plk1 molecules are densely loaded into ARGN by hybridizing with one functional region (called S-container) of RCA-p, obtaining the S-ARGN. Dox species can be efficiently encapsulated via intercalating into GC-rich regions, leading to the generation of DS-ARGN cluster bomb. The corresponding full name is Dox/SiRNA-encapsulated (DS) Aptamer-guided (A) nanocluster (N) bomb assembled by bundling gold nanorods (G) with rolling circle amplification product tape (R). The drug loading capacity for both siRNA and chemotherapeutics is increased by more than two orders of magnitude, and aptamer-mediated tumor cell-targeting ability can remain unchanged for a long time in a complex biological environment due to the internal compensation of degraded aptamers on the external surface originating from the structural reconfiguration of ARGN. The highly-resistant ARGN efficiently inhibits the leakage of loaded drugs during blood circulation and prevents the loaded siRNAs from enzymatic degradation, accomplishing the precision delivery of chemotherapeutic agents and gene drugs to diseased tissues of tumor-bearing mice and achieving desirable aptamer-promoted cellular internalization. Within cancerous cells, the cluster bombs can eject the different lethal bomblets, inducing the death of target cells. Namely, intracellular stimuli-responsive release of siRNAs and Dox species contribute to the gene therapy and chemotherapy, respectively. Moreover, AuNR nanocluster possesses high photothermal conversion efficiency and thus causes desirable PTT outcomes upon the NIR laser irradiation. As a result, the synergistic combination treatment of the three treatment modes is obtained. The *in vivo* therapeutic experiments against nude mice bearing human HeLa tumor xenografts demonstrate that DS-ARGN bomb not only 100 % suppresses the tumor growth but also causes negative growth in tumor volume and no systemic toxicity is detected, manifesting the potential tool for precision cancer therapy.

## 2 Results and discussion

### 2.1 Design of DS-ARGN cluster bomb and working mechanism for multimodal precision cancer therapy

**Scheme 1:**
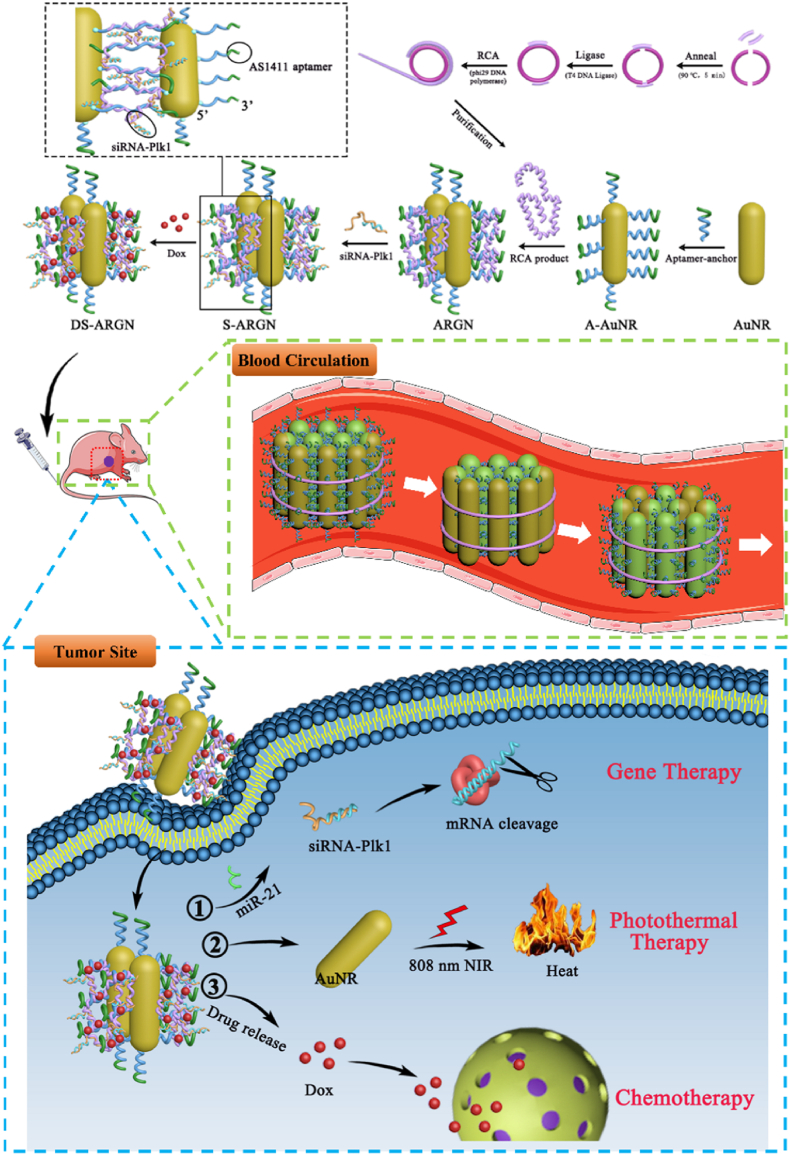
**Schematic illustration of the construction of DS-ARGN bomb and multimodal cancer therapy *in vivo*.** The preparation procedure of S-ARGN carrier is outlined as follows: modifying gold nanorod (AuNR) with Apt-anchor, bundling of AuNRs by RCA-p and loading siRNA-Plk1. After loading chemotherapeutics Dox into ds-DNA layer, the dual-drug-loaded S-ARGN (DS-ARGN) bomb is intravenously administrated into tumor-bearing animal model. During traveling a long and arduous journey via blood circulation, DS-ARGN bomb undergoes structural reconfiguration, can withstand the degradation by nuclease and retain its targeting capability, thus actively accumulating in tumor site, ejecting three different types of destructive bomblets and achieving desirable synergistic therapeutic effects of RNA interference therapy, chemotherapy and photothermal therapy of AuNR upon 808-laser irradiation.

Serum stability and tumor targeting capability are two main challenges for the delivery of anticancer therapeutics, especially siRNAs. In this study, the conceptual design of nucleic acid nanodevice DS-ARGN with multiple functional elements comes from the cluster bomb made of many explosive bomblets and is shown in Scheme 1. The Apt-anchor-functionalized gold nanorods (A-AuNR) are bundled together by RCA product, forming the reconfigurable cell-targeting aptamer-contained nanocluster of gold nanorods available for the loading of siRNAs and doxorubicin (Dox, a chemotherapeutic agent) in its interior domain. Such a glomeroplasmatic structure is resistant to enzymatic degradation and can itself ‘present’ aptamer molecules from the internal to its surface to compensate for the consumption induced by unwanted degradation, thereby holding the *in vivo* persistence of tumor targeting ability and offering the protection of encapsulated siRNAs against endogenous nuclease attack. Specifically, firstly, a circular padlock was prepared by ligation-based cyclization of two linear DNA strands (Circle-1 and Circle-2) with two short templates (Template-1 and Template-2). RCA reaction was performed using Template-1 or Template-2 as the primers in the presence of phi29 DNA polymerase, generating a long single-stranded DNA product of head-to-tail tandem repeats (RCA-p). The base sequence of structural unit of RCA product (SUR) is shown in Table S1. Within each SUR unit, two same fragments (TS-fragments) can perfectly hybridize with the miRNA-21, a potent oncomiR over-expressed within most cancers [47,48] and also is partly complementary to the extended part of Plk1-sense, while the other two domains going in the opposite directions (TO-fragments) are designed to be capable of hybridizing with the two fragments in the extended part of Apt-anchor. The 3′ end of Apt-anchor contains an AS1411 aptamer as a cancerous tissue-specific recognition functional region via binding to an over-expressed membrane receptor nucleolin [49] and the 5′ end is labeled with a mercapto group. To achieve the tumor-targeting capability, gold nanorod (AuNR) was covalently modified with Apt-anchor through well-established dative covalent thiol-Au bonds [50,51], producing an aptamer-anchor functionalized gold nanorod (A-AuNR). Then, A-AuNR was mixed with RCA-p and incubated with Plk1-antisense/Plk1-sense (siRNA-Plk1), forming siRNA-encapsulated (S) Aptamer-guided (A) nanocluster (N) assembled by bundling gold nanorods (G) with RCA-p tape (R) (abbreviated as S-ARGN). The possible molecular mechanism for the interaction between RCA-p and AuNR-confined Apt-anchors is described in Scheme S1. Theoretically, each SUR unit can load two siRNA-Plk1s and bind to two different Apt-anchors due to the spacer between TO-fragments. Because RCA-p is a long ssDNA stand made of many repeated structural units and can interact with Apt-anchors confined on different surfaces, it figuratively serves as a bundling adhesive tape and ties several Au nanorods together. Afterwards, Dox is loaded into the double-stranded fragments formed mainly from hybridization of RCA-p with AuNR-confined Apt-anchors via intercalating into GC base pairs [[52], [53], [54], [55]], leading to the formation of DS-ARGN cluster bomblet.

When exerting therapeutic treatment, once intravenously administrated into tumor-bearing living organisms, because various supplies are shipped to various living cells via the blood circulation [56], DS-ARGN bomb can reach any target organ or tissue and specifically accumulate in tumor issues. Since a substantial quantity of RCA-p sequences assemble to form a nucleic acid shell around S-ARGN and AuNRs have large steric hindrance, this structure can effectively safeguard the internal siRNA against degradation [[57], [58], [59]]. During traveling a long and arduous journey *in vivo*, although the Apt-anchors arranged on the outside surface of ARGN might be partly degraded by endogenous nucleases, the interior Apt-anchors could be exposed to the surrounding environment due to the structural reconfiguration of DS-ARGN (Scheme S2), offering the compensation for the loss of degraded aptamers. This can allow DS-ARGN possess a persistent tumor targeting ability and more efficiently accumulate in tumor sites. once internalizing into cancerous cells, different therapeutic drugs are ejected from main compartment and expectably exert synergistic therapeutic effects of three therapeutic modalities. ① Gene therapy. Due to the interaction of DS-ARGN with intracellular miR-21, the loaded-siRNA-Plk1 is released by competitive hybridization and silencing the plk1-mRNA expression, causing the G2-M phase cell cycle arrest and cell apoptosis [60]. ② Photothermal treatment. Upon near-infrared laser irradiation, AuNR can converse the optical energy into local heat and induce the thermal ablation of cancerous cells and tissues [61,62]. ③ Chemotherapeutic treatment. Dox is released upon the intracellular near-infrared radiation-induced temperature increase and enzymatic degradation of RCA-p tapes, thereby causing tumor cell apoptosis [63].

### 2.2 Construction and structural characterization of S-ARGN

**Fig. 1:**
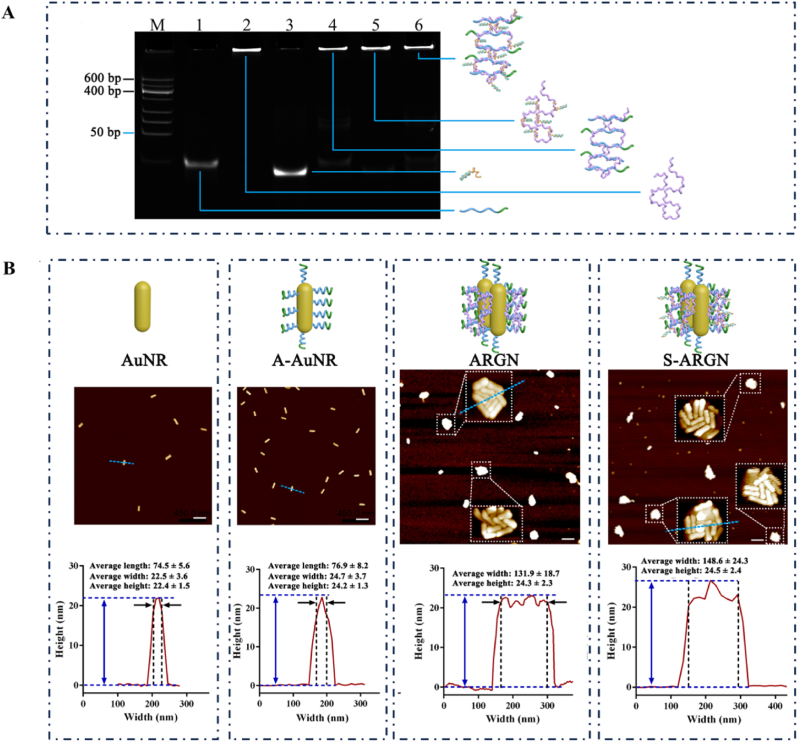
**Construction and characterization of S-ARGN. (A)** 15 % nPAGE characterization of assembly of S-AFR (siRNA-encapsulated (S) aptamer-folded (AF) rolling circle amplification tape (R)). Lane 1: Apt-anchor; Lane 2: RCA-p; Lane 3: D-siRNA-Plk1 (D-Plk1-sense + D-Plk1-antisense); Lane 4: RCA-p + Apt-anchor; Lane 5: RCA-p + D-siRNA-Plk1; Lane 6: S-AFR (RCA-p + Apt-anchor + D-siRNA-Plk1). The concentration of RCA-p is 2 μM. The concentrations of Apt-anchor, D-Plk1-sense and D-Plk1-antisense are 500 nM. M means low molecular weight DNA marker. **(B)** Atomic force microscopy (AFM) images of S-ARGN and intermediate counterparts. The scale is 200 nm. The upper part is the schematic diagrams of different nanostructures, while the lower part shows the corresponding cross-section profiles alone the blue dotted line, accompanied by average width (AW) and average height (AH) (n = 20). (For interpretation of the references to color in this figure legend, the reader is referred to the Web version of this article.)

The generation of RCA-p was verified by PAGE analysis as described in Fig. S1. Compared with the bands in other lanes, DNA band in lane 7 is retarded at the initial position of gel well, indicating the formation of RCA-p. Fig. S2 describes the proportional relationship of the hybridization between Apt-anchor and RCA-p. Of note, the concentration of basic structural unit is used to represent the concentration of RCA-p. It can be found from panel A that, at the Apt-anchor-to-RCA-p ratio of 1:3.5, there is very small amount of residual Apt-anchor (lane 5), and no obvious Apt-anchor band is observed in Lane 6 (the molar ratio, 1:4.7). Similarly, as described in panel B, the hybridization ratio of D-siRNA-Plk1-to-the complex of Apt-anchor/RCA-p is about 1:2.8. The difference between the actual value and theoretical value of hybridization ratio should be attributed to the electrostatic repulsion between Apt-anchor (or D-siRNA-Plk1) and RCA-p with a partly-coiled configuration originating from its small secondary structure motif. When investigating the hybridization efficiency of individual step, the oligonucleotides (e.g., Apt-anchor) -to-RCA-p ratio of 4:1 was used, while the excessive amount of oligonucleotides was adopted for the preparation of DS-ARGN to achieve sufficient loading and the residual species were removed by centrifugation. Fig. S3 describes the efficient loading of D-siRNA-Plk1 into RCA-p and specific release by D-miR-21 (illustrated in Fig. S3A). As shown in Fig. S3B, the disappearance of D-siRNA-Plk1 band in lane 5 indicates the efficient loading of D-siRNA-Plk1 into RCA-p, while appearance of D-siRNA-Plk1 band in lane 6 in the presence of D-miR-21 implies the release of D-siRNA-Plk1 from R-DS. Moreover, other family member counterparts cannot compete for D-siRNA-Plk1 species with RCA-p as shown in lanes 4, 5 and 6 of Fig. S3C. Fig. 1A describes the simultaneous hybridization of Apt-anchor and D-siRNA-Plk1 with RCA-p. Besides the disappearance of Apt-anchor band in lane 4 and D-siRNA-Plk1 band in lane 5, the simultaneous disappearance of the two bands in lane 6 demonstrate that RCA-p simultaneously hybridizes with Apt-anchor and D-siRNA-Plk1.

The morphology and size of AuNR and subsequently-modified ones were firstly examined by atomic force microscope (AFM). As shown in Fig. 1B, compared with naked AuNR, A-AuNR show a slightly larger size, including the larger length, width and height, but the rod-shaped morphology remains unchanged, indicating the immobilization of Apt-anchors on the surface. Once RCA-p is introduced, AFM image evidences that several AuNRs are indeed pulled together and form ARGN cluster, implying that RCA-p can serve as an adhesive tape and bundle the separate A-AuNRs. The average number of AuNR in each ARGN is 10 according to the statistical analysis (n = 10). The average width (AW) and average height (AH) of ARGN are 131.9 ± 18.7 nm and 24.3 ± 2.3 nm, respectively. The loading of D-siRNA-Plk1 (abbreviated as S) into ARGN only leads to the slight increase in the size (corresponding product, S-ARGN, with the AW of 148.6 ± 24.3 nm and AH of 24.5 ± 2.4 nm), which results from the D-siRNA-Plk1-induced volume expansion. Fig. S4A (TEM image) in verifies that AuNRs have the rod-shaped morphology and uniform size distributions and Fig. S4B displays that AuNRs exhibit the classical UV–vis spectral features, while Fig. S5 further validates the formation of AuNR nanoclusters upon the bundling by RCA-p nanotape via transmission electron microscope (TEM). Moreover, each AuNR unit of S-ARGN was estimated to averagely have 206 Apt-anchor molecules (Fig. S7), 167 RCA-p tapes (estimated from the number of structural unit) (Fig. S8) and 282 siRNA-Plk1 species (Fig. S9), indicating that all structural units of RCA-p are loaded with siRNA-Plk1s (100 % loading efficiency). Even taking into account that each structural unit has two binding sites for loading siRNA-Plk1s, the utilization efficiency of binding sites of RCA-p is more than 84 % ([282/(167 × 2)] × 100 %). Taking into account that each ARGN contains10 AuNR units on average, the siRNA loading content of ARGN is up to 2820, indicating 188-fold higher enhancement of loading capability than literature value [64]. It is important to note that, as shown in Fig. S10, the ability of encapsulated siRNA-Plk1 to resist the enzymatic degradation is also substantially enhanced.

Subsequently, to verify the long-term stability of siRNA-Plk1 in ARGN, S-ARGN loaded with siRNA Plk1 was incubated with 10 % FBS at room temperature for 0 h, 12 h, 24 h, 48 h, and 72 h, followed by DLS measurement to evaluate the size change. As shown in Fig. S11A, the size of S-ARGN gradually decreases and no substantial change occurs within 24 h. Nevertheless, the hydrated particle size of S-ARGN is 85.6 ± 22.9 nm after 72 h, which was basically consistent with A-AuNR mentioned in Fig. 1, Fig. 5SA, indicating the dissociation into individual nanorods and the enzymatic degradation of DNA strands. The dPAGE analysis (Fig. S11B) shows that the residual amount of siRNA-Plk1 is 45 % at 24 h and 20 % at 48. The siRNA-Plk1still was not completely degraded at 72 h. These experimental data validate that S-ARGN does possess the long-term resistance to the enzymatic degradation.

### 2.3 Structural reconfiguration of ARGN based on a linear RCA-p as a flexible adhesive tape

Structurally, RCA-p is a long linear strand composed of many tandemly-repeated copies and can bundle A-AuNRs together via stringing the surface-confined Apt-anchors, contributing to the formation of cluster-shaped ARGN. Within ARGN, the interaction of RCA-p with A-AuNR is based on the common Watson-Crick base paring rather than covalent chemistry and thus ensures the sufficient flexibility to perform structural reconfiguration, allowing ARGN to easily adapt to environmental transient changes [65]. In this case, it is deemed that, when the exterior Apt-anchors on the surface of ARGN are degraded and net negative charge is reduced, ARGN reconfigures its own structure due to the internal tension originating from electrostatic repulsion and spatial expansion of compactly-folded DNA species, thereby presenting the interior aptamers onto the outside surface and thereby maintaining tumor-targeting ability. By comparing the AFM data (Fig. 1B), although the width of ARGN is much larger than A-AuNR, there is no substantial difference in the height, indicating that electrostatic interaction between the surface-confined DNA strands and mica substrate causes the ARGN nanocluster to collapse into a single layer structure [66]. TEM image also shows the structural collapse of ARGN and S-ARGN (Figs. S5B and S5C). Namely, the structural reconfiguration of ARGN indeed occurs, offering a unique opportunity for the interior aptamers to walk out onto the outside surface and thereby ensuring the persistent tumor cell-targeting ability in a complex biological environment.

To offer further evidence for the structural reconfiguration of ARGN nanocluster, a directly-related counterpart nanocluster C-ARGN was prepared by covalently crosslinking the individual AuNRs of ARGN with a DNA-based crosslinker (34 bases in length). As shown in Table S1, D-crosslinker is a doubly-thiolated DNA strand (D-crosslinker) in which two ends are modified with mercapto groups. It can simultaneously capture two different AuNRs via the thiol-Au linkage, generating a nonreconfigurable C-ARGN. The structural difference between ARGN and C-ARGN is described in Scheme S2. Because each of AuNRs is firmly fixed, once formed, the relative position of individual nanorods cannot be changed, which was verified by different microstructural characterization techniques. As shown in Fig. S6A, AFM image shows that C-ARGN also displays the nanocluster-based structure, which is further validated by TEM imaging (Fig. S6B). However, C-ARGN holds much lower average width (89.4 ± 12.2 nm, decreasing by 33.2 %) and significantly larger average height (42.4 ± 4.3 nm, increasing by 174.5 %) than ARGN. Namely, C-ARGN roughly maintains 3-dimensional configuration and has no substantial structural reconfiguration during the preparation of sample for AFM measurement compared with ARGN. Moreover, as shown in Fig. S6C, DLS data display that C-ARGN indeed has the smaller size than ARGN, implying a more compact structure. From the experimental results, it can be inferred that the internal tension of ARGN is substantially suppressed by the covalent crosslinking effect of D-crosslinker. Additionally, it should be noted that, if the long D-crosslinker strand was substituted with short 1,6-hexanedithiol that also has two mercapto groups at its two termini and is well-known to be able to trigger the formation of three-dimensional (3D) aggregate of gold nanoparticles [67] via crosslinking reaction [68], no obvious change in nanocluster size is detected compared with ARGN (not shown here). This is because hexanedithiol is too small to simultaneously capture two different Apt-anchor-functionalized gold nanorods in ARGN. The reasonable explanation is that there is a barrier between two adjacent AuNRs originating from the formation of DNA assembled materials on AuNR surface. Due to the structural reconfiguration, the tumor-targeting capability of ARGN nanocluster exhibits the appreciable tolerance to a complex biological environment.

### 2.4 Specific recognition of S-ARGN toward tumor cells

**Fig. 2:**
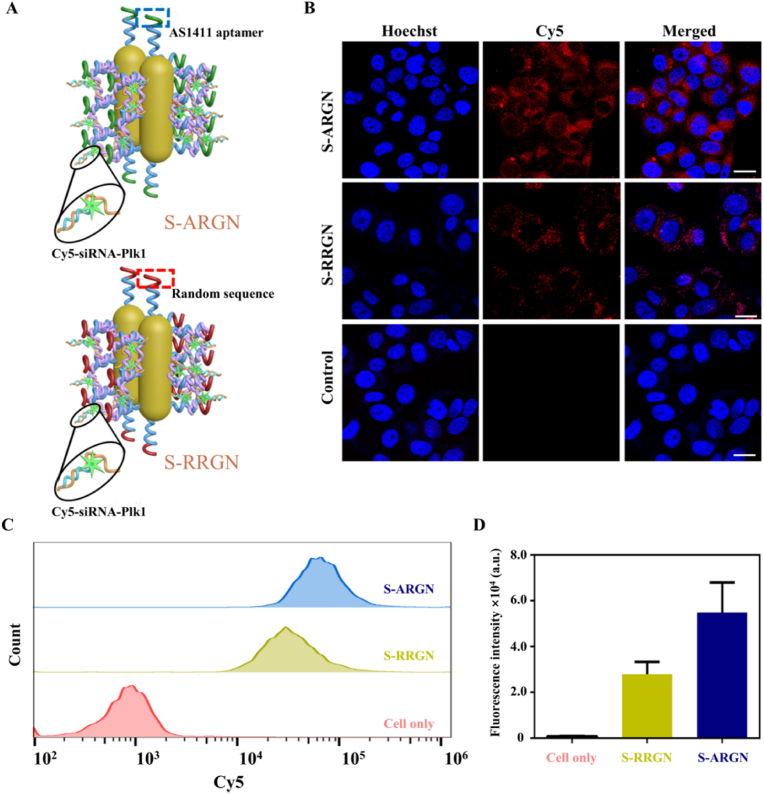
**Aptamer-dependent cell-recognition specificity of S-ARGN towards MCF-7 cells. (A)** Schematic diagram of S-ARGN and counterpart S-RRGN (substituting Apt-anchor with a random sequence, R-anchor), where the enlarged Cy5-siRNA-Plk in indicated in solid black circles. In the dotted color box, the green terminal fragments represent AS1411 aptamer, while the red terminal fragments is random DNA sequence. **(B)** Confocal fluorescence images of MCF-7 cells treated with S-ARGN, S-RRGN or PBS for 4 h. The blue regions indicate the nuclei stained by Hoechst 33342. The scale is 20 μm. **(C)** Flow cytometry analysis of MCF-7 cells treated with S-ARGN, S-RRGN or PBS. **(D)** Quantitative analysis of fluorescence intensity of the samples involved in panel C. The data are displayed as means ± SD (n = 3). The concentration of Cy5-S-ARGN is 48 pM. S-RRGN is a counterpart where AS1411 aptamer is substituted with a random sequence. (For interpretation of the references to color in this figure legend, the reader is referred to the Web version of this article.)

Precisely targeting cancer cells is required for anti-cancer drug nanocarriers to mitigate the damage to normal cells and improve the therapeutic effect. In order to make S-ARGN have the ability to selectively target cancer cells, AS1411 aptamer was used as a specific recognition element because it can recognize the nucleolin overexpressed on the surface of a variety of cancer cells [69,70], including MCF-7 cells and HeLa cells [71]. In order to examine whether AS1411 aptamer can play a targeting role in the cellular internalization of S-ARGN, a counterpart, siRNA-encapsulated (S) random sequence-guided (R) nanocluster (N) assembled by bundling gold nanorods (G) with rolling circle amplification tape (R) (abbreviated as S-RRGN), was constructed by substituting AS1411 aptamer with a random sequence, and Cy5-Plk1-antisense was used for the preparation of siRNA-Plk1 to offer fluorescence signal output. The structural similarities and differences between S-ARGN and S-RRGN are described in Fig. 2A, where Cy5 fluorescence is quenched via the fluorescence resonance energy transfer (FRET) effect by AuNR. The confocal fluorescence images of MCF-7 cells treated with S-ARGN, S-RRGN and PBS (control) are shown in Fig. 2B. It is found that Cy5 fluorescence intensity of S-ARGN group is much higher than both S-RRGN and PBS, which is also validated by flow cytometric analysis (Fig. 2C and D). However, for normal human hepatocyte (L02) cells, a nucleolin-negative cell line, not only is the fluorescence intensity substantially compromised but also there is no obvious difference between the groups of S-ARGN and S-RRGN (Fig. S12), indicating the aptamer sequence-dependent internalization of S-ARGN into cancer cells.

### 2.5 Stimuli-responsive release of encapsulated siRNAs and high RNAi efficacy

**Fig. 3:**
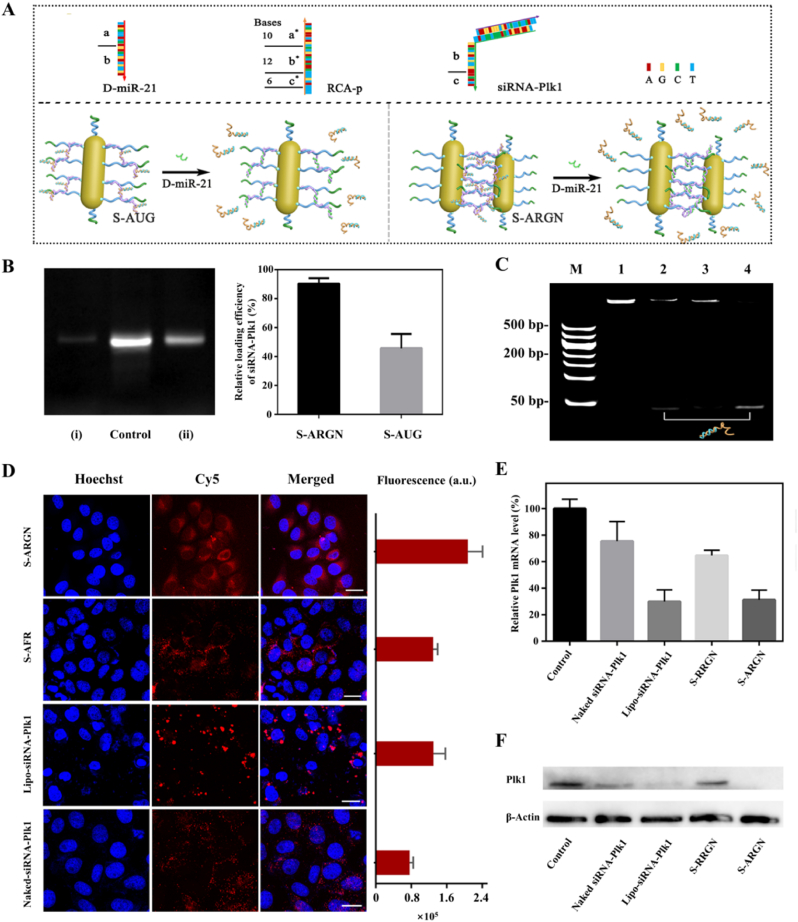
**The efficacy of S-ARGN for the targeted delivery of siRNA-Plk1 to cancer cells. (A)** Schematic diagram of the release of siRNA-Plk1 in a miR-21 stimuli-responsive fashion from S-ARGN or its counterpart S-AUG. **(B)** 15 % nPAGE analysis of siRNA-Plk1-loading efficiency of ARGN: siRNA-Plk1 remaining in the supernatant after centrifugation of S-ARGN (i) and S-AUG (ii). The control group is the equal amount of free Cy5-siRNA-Plk1 composed of Cy5-Plk1-antisense and D-Plk1-antisense. The fluorescence intensity of gel bands was analyzed by imagej software. The relative loading efficiency of siRNA-Plk1 was calculated by formula (F-F_R_)/F × 100 %, where F represents the fluorescence intensity of Control group and F_R_ denotes the fluorescence intensity of siRNA-Plk1 remaining in the supernatant after centrifugation S-ARGN or S-AUG. **(C)** 15 % nPAGE imaging to verify the release of siRNA-Plk1 through the toehold-mediated strand displacement reaction. Lane 1: S-AUG; Lane 2: S-AUG + D-miRNA-21; Lane 3: S-ARGN; Lane 4: S-ARGN + D-miRNA-21. **(D)** Evaluation of the ability of S-ARGN to execute the cancer cell-targeted siRNA-Plk1 delivery by comparative analysis using HeLa cells as cell models and Cy5-siRNA-Plk1 was used to offer a fluorescence signal. S-AFR is composed entirely of nucleic acids (siRNA-Plk1, aptamer and RCA-p) without AuNRs. Lipo-siRNA-Plk1 indicates the cellular internalization of siRNA-Plk1 through cationic liposome-mediated transfection. Naked siRNA-Plk1 implies the incubation of siRNA-Plk1 with cells without any ancillary species, such as AuNR and transfection reagents. The histogram on the right shows the quantitative analysis of intracellular Cy5 fluorescence intensity. The content of siRNA-Plk1 in each group was consistent. The scale is 20 μm. **(E)** Quantitative analysis of Plk1 mRNA levels by qPCR. Data are displayed as means ± SD (n = 3). **(F)** Western blot analysis to evaluate the inhibition of PLK1 protein expression by S-ARGN and other counterparts. The equivalent concentration of siRNA-Plk1 was involved in each group. For the control group, siRNA-loaded nano-formulation was substituted with PBS.

In order to explore whether RCA-p is beneficial to improve the loading efficiency of siRNA-Plk1, we constructed a counterpart formulation, S-AUG, SiRNA -encapsulated (S) aptamer-guided (A) gold nanorods (G) modified by the structural unit of rolling circle amplification product (U). As described in Fig. 3A, whether in S-ARGN and S-AUG, miR-21 can compete with siRNA-Plk1 for the binding site designed in the structural units of RCA-p because of its higher binding affinity originating from more complementary bases. The same amount of Cy5-siRNA-Plk1s was employed for the preparation of S-ARGN and S-AUG, and the loading efficiency to siRNA-Plk1 was estimated by measuring the residual amount of Cy5-siRNA-Plk1 in the supernatant upon centrifugation by nPAGE analysis. As shown in Fig. 3B, it can be found that the brightness of residual Cy5-siRNA-Plk1 band of Group-i (S-ARGN) is much lower than S-AUG, indicating that ARGN has substantially larger siRNA cargo loading capability. If the amount of Cy5-siRNA-Plk1 in the control group is defined as 100 %, the relative loading capabilities of S-ARGN and S-AUG are 90 % and 43 %, respectively. Presumably, for ARGN composed of multiple gold nanorods and long RCA-p wires, only some structural units of RCA-p are required to hybridize to the surface-confined Apt-anchors and the other structural units are suspended. Thus, ARGN exhibits the reduced rigidity and relatively weak electrostatic repulsion, promoting the interaction of siRNA cargos with binding sites. Possibly for the same reason, the loaded siRNA-Plk1s are almost 100 % released from S-ARGN as seen in Fig. 3C. No obvious residual DNA band is detected in Lane 4 upon the addition of D-miRNA-21, which contrasts sharply with S-AUG (lane 2).

In order to verify whether the release of siRNA-Plk1 is miR-21-dependent process or not, MCF-7 cells was pretreated with estradiol (E_2_) to inhibit the miR-21 expression [[72], [73], [74]] and then treated with Cy5-labeled S-ARGN, followed by confocal fluorescence imaging. As shown in Fig. S13A, compared with native MCF-7 group, the Cy5 fluorescence signal of MCF-7-E_2_ group is considerably reduced, suggesting the limited miR-21 release. Fig. S13B shows that the relative expression level of miR-21 within E_2_-treated MCF-7 cells is only 43.5 % compared with native MCF-7 cells, implying the amount of released siRNA-Plk1 is indeed determined by the miR-21 expression level. Moreover, HeLa cells were also incubated with S-ARGN and fluorescently imaged under identical conditions, and the experimental results are presented in Fig. S13C. One can found that a lower fluorescence signal is detected, which is consistent with the lower miR-21 expression level reported by literature studies [[75], [76], [77]]. The specific stimuli-responsive release of siRNA-Plk1 is expected to be conducive to improving the efficacy of gene therapy and reducing the toxicity to normal cells. Moreover, as shown in Fig. 3D, the cellular internalization efficiency of siRNA-loaded formulation is considerably suppressed in the absence of A-AuNR (S-AFR group), and naked siRNA-Plk1 exhibits the lower uptake. Interestingly, S-ARGN has the higher efficiency in delivering siRNAs than commercial transfection reagents (Lipo-siRNA-Plk1 group).

The therapeutic outcome of ARGN-mediated RNAi therapies was first explored at the cellular level by estimating the expression level of PLK1 mRNA and PLK1 protein [78,79]. For this purpose, HeLa cells were with siRNA-Plk1-packaged AuNR nanocluster (S-ARGN) for 24 h and then expression level of Plk1 mRNA was estimated via real-time PCR. Fig. 3E shows that S-ARGN indeed efficiently inhibits the expression of Plk1 mRNA. The slightly lower relative expression of Plk1 mRNA in the Lipo-siRNA-Plk1 group than S-ARGN is possibly because the small steric hindrance of free double-stranded siRNA makes it to interact with RNA-induced silencing complex (RISC) more easily and degrade target mRNA more rapidly. However, Western Blot analyses show that PLK1 protein band of S-ARGN group almost completely disappears, and the band brightness is lightly lower than Lipo-siRNA-Plk1 group (Fig. 3F), indicating a more desirable gene silencing activity. This should be attributed to the sustained gene silencing effect originating from the protection of siRNA-Plk1 by S-ARGN against intracellular enzymatic degradation [80]. The confocal fluorescence image in Fig. S14 further proves the protective effect of S-ARGN on siRNA-Plk1.

### 2.6 Photothermal therapeutic performance

**Fig. 4:**
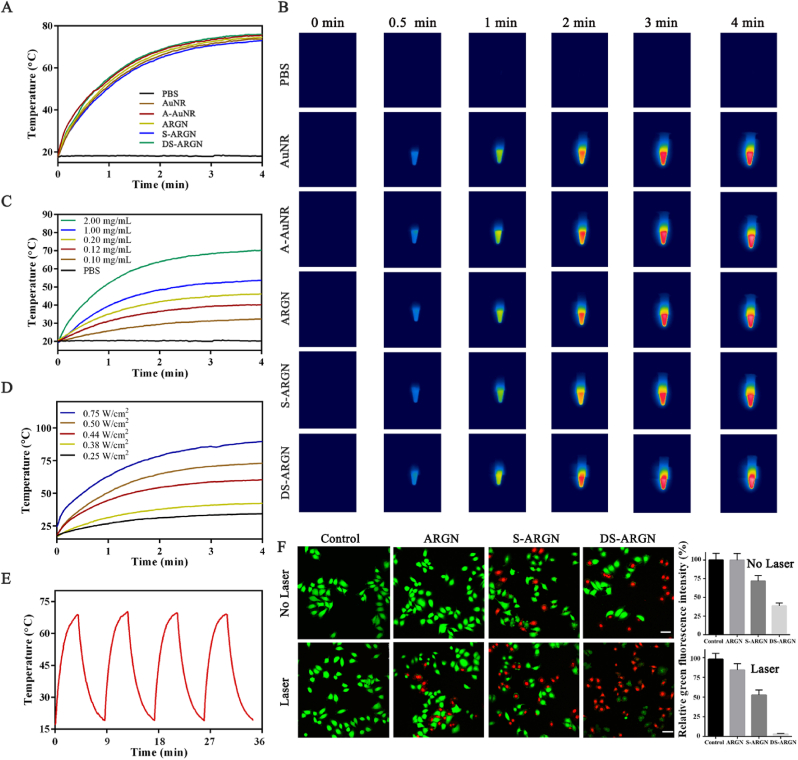
**Near infrared (NIR) photothermal performance of DS-ARGN**. **(A)** Temperature increase curves of DS-ARGN (2 mg/mL) and its counterparts in PBS dispersion under 808 nm laser irradiation at a power intensity of 0.5 W/cm^2^ for 4 min. **(B)** Representative near infrared (NIR) thermal images of the samples in panel A at different time points. **(C)** Temperature increase curves of DS-ARGN in PBS dispersion at different concentrations (0.10, 0.12, 0.20, 1.00 and 2.00 mg/mL) under 808 nm laser irradiation at 0.5W/cm^2^ for 4 min. **(D)** Temperature increase curves of DS-ARGN in PBS dispersion (2.00 mg/mL) under 808 nm laser irradiation at various laser intensities (0.25, 0.38, 0.44, 0.50, 0.75 W/cm^2^) for 4 min. **(E)** Heating and cooling cycles of DS-ARGN solution (2.00 mg/mL) under 808 nm laser irradiation (0.50 W/cm^2^). **(F)** Confocal fluorescence images of HeLa cells exposed to the photothermal therapy with near-infrared radiation, accompanied by relative fluorescence intensity (RFI) to quantitatively determine the cell viability. For the control group, PBS was instead administrated. RFI value of control group is defined as 100 %. Calcein-AM can stain live cells and appear green, while PI can stain dead cells and appear red. The equivalent concentrations of Dox and siRNA-Plk1 are 0.6 μM and 0.1 μM, respectively. The scale is 50 μm. (For interpretation of the references to color in this figure legend, the reader is referred to the Web version of this article.)

The photothermal performance of S-ARGN was investigated in order to evaluate the therapeutic effects of photothermal therapy (PTT). As shown in Fig. 4A, the temperature of different samples under laser irradiation (808 nm, 0.5 W/cm^2^) was monitored. The temperature of PBS almost remains unchanged over the entire examination period of 4 min, while the temperature of five samples containing AuNR increases significantly (from 17.5 °C to 75 °C) within 4 min, which is much higher than the lethal temperature (42 °C) of tumor cells [81]. Moreover, there is no obvious difference in the temperature change between the five samples. The infrared thermal images of corresponding samples are shown in Fig. 4B. The experimental results demonstrate that AuNR used in this study has good photothermal performance, and DNA modification, bundling together with RCA-p and loading with gene drugs and chemotherapeutic agents do not affect the photothermal conversion efficiency of AuNR.

**Fig. 5:**
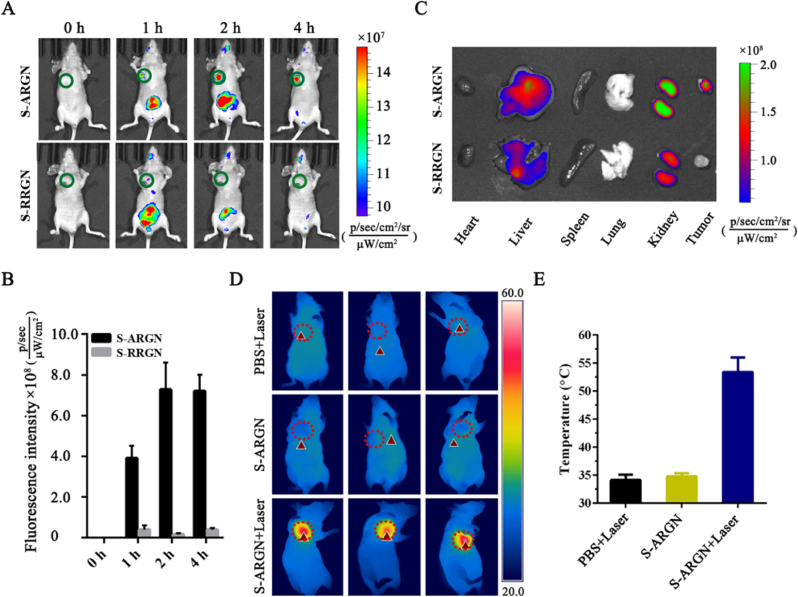
**Superior tumor-targeting ability of S-ARGN vehicle *in vivo*. (A)** Fluorescence images of nude mice bearing HeLa tumor administrated separately with S-ARGN and its counterpart S-RRGN via whole animal imaging modalities. S-ARGN or S-RRGN was intravenously injected into the tail vein of living tumor-bearing nude mice, and the fluorescence imaging was performed at different time intervals (1 h, 2 h and 4 h). The tumor sites are marked in the dark green circle. **(B)** Quantitative analysis of fluorescence intensity emitting from tumor sites corresponding to panel A. The image of mice before administration of formulations is used as the point of 0 h. **(C)** The *ex vivo* images of organs and tumor tissues harvested from mice at 4 h post-administration of nano-formulations. The images of panels A and C and fluorescence data were acquired by FOTRIC AnalyzIR software. **(D)** NIR thermal imaging of nude mice bearing HeLa tumor at 2 h post-injection of S-ARGN and controls under the irradiation of 808 nm laser (0.5 W/cm^2^, 1 min). Each group includes three tumor-bearing mice. **(E)** The temperatures measured at tumor sites of mice in panel D. (For interpretation of the references to color in this figure legend, the reader is referred to the Web version of this article.)

To explore whether the photothermal conversion performance of AuNR is controlled, the temperature changes of different ARGN-based samples were monitored under different experimental conditions. Fig. 4C shows the dynamic change of the temperature of S-ARGN solution at different concentrations under given laser irradiation (808 nm, 0.5 W/cm^2^). One can notice that the temperature increase rate increases with the increment of S-ARGN concentration and the larger S-ARGN concentration, the higher the final temperature, indicating that the accumulation of S-ARGN in the diseased sites is conducive to PTT efficacy. When the lasers of different powers (0.25 W/cm^2^, 0.38 W/cm^2^, 0.44 W/cm^2^, 0.50 W/cm^2^ and 0.75 W/cm^2^) were used to irradiate S-ARGN, the temperature of solutions change accordingly (Fig. 4D). The larger laser power density, the higher the solution temperature. Moreover, as an important factor for the effective killing of tumor cells [82], the photothermal stability of S-ARGN was tested by monitoring the temperature changes when performing four repetitive cycles of laser irradiation (the power density, 0.5 W/cm^2^) between “on” and “off”. As shown in Fig. 4E, during the four cycles of heating and cooling, there is no detectable change in the highest temperature and lowest temperature between different temperature cycles, indicating that S-ARGN has the desirable photothermal stability.

### 2.7 High loading capability of anti-cancer drugs and targeted delivery

Doxorubicin (Dox), as an anticancer chemotherapeutics widely used in the treatment of various cancers, can be loaded into various DNA structures by intercalating into the G-C base pairs of DNA double strands [55,[83], [84], [85]]. Dox kills tumor cells and suppresses *in vivo* tumor growth by entering the nucleus and inhibiting DNA replication [86]. Following the exploration of the ability of S-ARGN to exert the targeted delivery of siRNA-Plk1 and exhibit photothermal conversion efficiency, the performance of S-ARGN in delivering Dox was evaluated. The concentration of Dox has a good linear relationship with the fluorescence intensity at 560 nm (Fig. S15). Because of the fluorescence quenching caused by the intercalation of Dox into the G-C base pairs in the DNA double-strand [54], the fluorescence intensity of Dox is gradually weakened as the concentration of S-ARGN increases (Fig. S16A), confirming the efficient loading of Dox into S-ARGN. The corresponding dynamic relationship between the concentration of S-ARGN and the concentration of actually-loaded Dox was quantitatively estimated as shown in Fig. S16B. The data at the turning-point show that one AuNR can carry 1586 of Dox molecules, and one ARGN can accommodate 15860 Doxs since each ARGN is composed of 10 AuNRs on average, indicating the 24∼317-times improved drug loading capability [3,87]. Moreover, as shown in Fig. S16C, DS-ARGN has no unintended drug leakage even under shaking at 200 rpm and 37 °C for 12 h, but the laser irradiation causes the efficient release of Dox under identical conditions. In addition, compared with DS-RRGN without AS1411 aptamer, MCF-7 cells treated with AS1411-functionalized DS-ARGN show much stronger fluorescence of Dox (Fig. S17A), indicating that Dox can be specifically transported into target tumor cells by combining with cell-targeting aptamer. The flow cytometric analysis confirms the same experimental results (Figure S17B, Figure S17C).

### 2.8 Synergistic anti-cancer efficacy of multiple therapeutic modalities in vitro

Targeted multimodal synergistic cancer therapy based on the combination of gene therapy, photothermal therapy, and chemotherapy was systematically evaluated in this section. The left half of Fig. 4F describes the CLSM images of calcein-AM/PI double stained HeLa cells treated with different drug-loaded formulations, where the calcein-AM green fluorescence indicates the living cells, while propidium iodide (PI) red fluorescence denotes the dead cells. The right half represents the corresponding quantitative estimation of living cells based on the green fluorescence intensity. It can be noticed that, for Control group, no dead cell is observed regardless of laser irradiation. For the other three groups, more dead cells (red) are detected when subjected to laser irradiation. Moreover, compared with the single photothermal therapy (ARGN), the combination of photothermal therapy with gene therapy (S-ARGN) causes the increase in the number of dead cells. The triple-modal combinatorial therapy (DS-ARGN) composed of gene therapy, photothermal therapy, and chemotherapy exhibit the highest antitumor efficacy because the highest number of dead cells appear, indicating great promise in targeted cancer therapy.

Moreover, to systematically evaluate the multimodal combinational therapy, the proliferation of HeLa cells was tested by CCK-8 assay upon the treatment with the different concentrations of various drug formulations. As shown in Fig. S18, ARGN alone does display detectable cell cytotoxicity, and the single-modal therapy strategies, including chemotherapy (D-ARGN), gene therapy (S-ARGN) and photothermal therapy (ARGN + laser) alone, can obviously inhibit cell proliferation. The dual-modal combinatorial therapies, including RNAi-/photothermal therapy (S-ARGN + Laser), chemo-/photothermal therapy (D-ARGN + Laser) and RNAi-/Chemotherapy (DS-ARGN), cause the further decrease of cell viability, indicating more desirable therapy efficacy. The strongest cytotoxicity is obtained upon the triple-modal combinatorial therapy (DS-ARGN + Laser), and the corresponding cell survival rate is only 27.0 %, indicating the substantially improved synergistic therapeutic outcomes.

In addition, the materials themselves (S-ARGN-D), where siRNA-Plk1 was substituted with DNA analogue (D-siRNA-Plk1), no Dox was involved and laser irradiation was not used, have no obvious cellular cytotoxicity regardless of cell nature. As shown in Fig. S19, the lowest cell viability of L02 cells is 89.6 %, which is reasonable because the as-assembled materials cannot enter L02 cells (Fig. S12). Even if assembled materials can be efficiently internalized into MCF-7 cells (Fig. 2), the cell viability of MCF-7 cells treated with the highest concentration of S-ARGN-D is more than 85 %, indicating the desirable biocompatibility.

### 2.9 *In vivo* anti-tumor activity of DS-ARGN

Firstly, the *in vivo* dynamic distribution of carriers alone was monitored in a real-time fashion to evaluate the ability of S-ARGN to target tumor sites. For this purpose, S-ARGN labeled with Cy5 was systemically administrated into HeLa tumor-bearing BALB/c nude mouse models via single tail vein injection, followed by CLSM imaging after a certain period of time using an IVIS small animal imaging station. The Cy5-S-RRGN was intravenously administrated into mice under identical conditions and used as control. As shown in Fig. 5A, for S-ARGN group, the red fluorescence signal appears in tumor site at 1 h post-administration, increases to a very high value at 2 h and still remains strong at 4 h although the red color fluorescence in the abdomen is almost completely faded, indicating the accumulation of S-ARGN in cancerous issues and persistent existence. In contrast, for S-RRGN group, no obvious fluorescence is observed in tumor site. Fig. 5B shows the quantitative fluorescence intensity emitting from tumor sites. At 4 h post-injection, nude mice were sacrificed, and tumor tissues and other major organs were harvested for *ex vivo* fluorescence imaging. As shown in Fig. 5C, a strong fluorescence signal emits from tumor site in S-ARGN group, while there is no observable fluorescence signal in tumor site in S-RRGN group. Meanwhile, the high fluorescence signal is also observed in liver and kidney, which reasonable because they are the two major excretory organs of the body [88]. Moreover, to examine whether S-ARGN exhibits excellent *in vivo* photothermal performance, we injected S-ARGN into BALB/c nude mice bearing subcutaneously inoculated HeLa tumors (about 100 mm^3^) through tail vein. After 4 h post-administration, 808 nm laser was used to irradiate tumor site for 1 min, and then infrared thermal imaging was performed immediately. From Fig. 5D, one can notice a significant temperature increase in tumor site upon laser irradiation, and the temperature is as high as 53.4 °C (Fig. 5E), which is much higher than the lethal temperature of tumor cells [55].

**Fig. 6:**
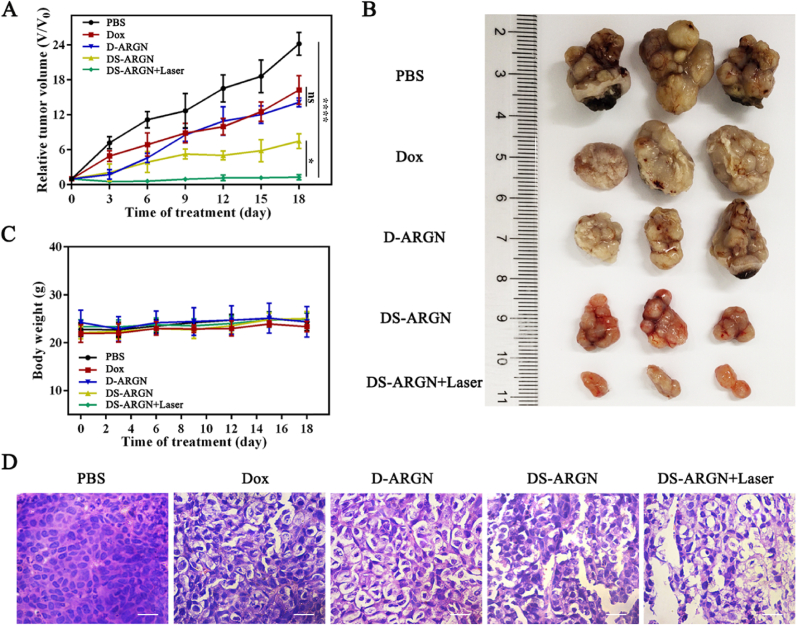
**Synergistic therapeutic efficacy of DS-ARGN in murine tumor models. (A)** The change in relative tumor volume measured *in vivo* throughout the period of treatment (up to 18 days). The relative tumor volume was calculated by V/V_0_, where V_0_ represents the tumor volume before the first treatment (day 0), and V indicates the tumor volume measured after each treatment. ^ns^P>0.05, ∗P < 0.05, ∗∗∗∗P < 0.0001. **(B)***Ex vivo* photographs of tumors harvested from nude mice after 18 day post-treatment. **(C)** Body weight changes of tumor-bearing nude mice over the entire duration of treatment. **(D)** H&E-stained images of tumors harvested at 18 days post-treatment. The scale is 10 μm.

To explore the therapeutic efficacy of triple-modal combinatorial therapy *in vivo*, different drug-loaded formulations were separately administrated into tumor-bearing mice through tail vein every 3 days for 18 consecutive days. Before each administration, the tumor volume was measured with a vernier caliper, and the body weight was determined with an electronic balance. Moreover, after the tumor-bearing mice subjected to 18-day treatment were sacrificed, the tumors and major organs, including heart, liver, spleen, lung and kidney, were harvested for *ex vivo* fluorescence imaging and/or histological analysis with hematoxylin and eosin (H&E) staining. As shown in Fig. 6A, upon treatment with free Dox, the tumor volume slightly decreases, and D-ARGN-based treatment displays a slightly-higher therapeutic efficacy owing to the aptamer-mediated targeted drug delivery. The DS-ARGN-based simultaneous dual-modal therapy system can further promote the anticancer efficacy, showing the synergistic RNAi-/Chemotherapy that is comparable to the literature value [89]. This inhibited the tumor growth by about 70 %. Encouragingly, the combination of DS-ARGN therapy with laser irradiation (i.e., triple-modal combinatorial therapy) can almost 100 % suppresses the tumor growth. *Ex vivo* photographs of tumors harvested show the consistent experimental results (Fig. 6B): after subjected to the therapy based on the combination of DS-ARGN with laser irradiation, the tumor size is dramatically smaller than that corresponding to PBS group. No substantial differences in body weight between treatment groups were detected throughout the examination period (Fig. 6C), indicating that triple-modal combinatorial therapy is well-tolerated by mice. In addition, as described in Fig. 6D, the histological analysis of tumors shows that, compared with the PBS group, the tumors in the other groups have different degrees of damage. Among them, the tumors in the triple therapy group display the most serious damage so that the phenomena of cell shrinkage, nucleus condensation and extracellular space increment are most pronounced. Besides the substantially enhanced therapeutic outcomes, the histological analyses of H&E-stained slices of major organs harvested from tumor-bearing mice after 18-day treatment show the desirable systematic safety of triple-modal combinatorial therapy (Fig. S20). Specifically, Heart slices in free Dox treatment group show myocardial fiber breakage, which may be caused by the cardiotoxicity of Dox [90]. Other experimental groups do not show obvious damage to any organ mainly because AS1411 aptamers can persistently stand up onto the surface of DS-ARGN and ensure the precisely-targeted delivery of anticancer agents to specific tumor sites, thereby avoiding the off-target toxic side effects. Overall, the experimental results on *in vivo* anti-tumor activity demonstrate that DS-ARGN-mediated triple-modal therapy holds great potential as precision theranostic nanomedicines.

## 3 Conclusion

In this study, via using RCA product as a flexible tape, aptamer-functionalized gold nanorods (AuNRs) are bundled into a reconfigurable nanocluster (ARGN) that serves as drug carrier and photothermal conversion agents. After loading with chemotherapeutics and siRNAs, an intelligent powerful precise multimodal combinatorial therapy platform, DS-ARGN cluster bomb, is obtained for the RNAi-/Chemo-/PTT multimodal synergistic therapy. Several superior advantages of DS-ARGN bomb are described as follows: (i) Reconfigurable structure. Bundling AuNRs with a flexible RCA tape imparts a considerable degree of morphological flexibility to the finally-assembled nanocluster bomb, benefiting the maintenance of structural integrity in a complex biological medium. Thus, the internal cell-targeting ligands can move outwards for the compensation for the loss of surface-confined aptamers originating from enzymatic degradation, achieving a persistent tumor-targeting ability and ensuring the sufficient accumulation of therapeutic agents in tumor site even if administrated into living organisms; (ii) Enhanced resistance to nuclease degradation, which avoids the unintended drug leakage and the interference failure of siRNAs; (iii) High chemotherapeutics loading capability (15860 Doxs per ARGN), which guarantees the sufficient drug content in cancerous issues; (iv) High siRNA loading capability and well-controlled intracellular stimuli-responsive release, ensuring RNA interference efficiency and avoiding non-specific release of siRNAs; (v) High photothermal conversion efficiency, which makes laser irradiation cause the substantial increase in the temperature, thereby efficiently inducing cell apoptosis; (vi) Substantially-improved therapeutic efficacy. Specifically, the tumor growth *in vivo* can almost be completely inhibited by the triple-modal combinatorial therapy offered by DS-ARGN cluster bomb; (vii) Circumvention of off-target toxicity. The main normal organs have no detectable adverse cytotoxic side effects often encountered during the conventional chemotherapy. The DS-ARGN device shows great application potential in the precision cancer therapy and becomes an exemplary system on the basis of the combination of gene therapy, chemotherapy and photothermal therapy.

## CRediT authorship contribution statement

**Qian Gao:** Writing – original draft, Validation, Data curation, Conceptualization. **Weijun Wang:** Writing – original draft, Methodology, Conceptualization. **Shujuan Sun:** Project administration, Data curation, Conceptualization. **Ya Yang:** Investigation. **Kaili Mao:** Validation. **Yuxi Yang:** Conceptualization. **Zai-Sheng Wu:** Writing – review & editing, Validation, Supervision, Funding acquisition.

## Declaration of competing interest

The authors declare that they have no known competing financial interests or personal relationships that could have appeared to influence the work reported in this paper.

## Data Availability

Data will be made available on request.
