# Supplementary material for: Bundling gold nanorods with RCA-produced DNA tape into an intelligently reconfigurable nanocluster bomb for multimodal precision cancer therapy
Source: Mater Today Bio. 2025 Mar 28;32:101718. doi: 10.1016/j.mtbio.2025.101718 (PMC11999372; doi:10.1016/j.mtbio.2025.101718)
Supplement: Multimedia component 1 [file mmc1.docx]

**Supporting information**

**Bundling gold nanorods with RCA-produced DNA tape into an intelligently reconfigurable nanocluster bomb for multimodal precision cancer therapy**

Qian Gao, ^1,2#^ Weijun Wang,^1,2,3#^ Shujuan Sun,^1#^ Ya Yang,^1^ Kaili Mao,^2^ Yuxi Yang^2^ and Zai-Sheng Wu^1,2*^

^1^Cancer Metastasis Alert and Prevention Center, Fujian Provincial Key Laboratory of Cancer Metastasis Chemoprevention and Chemotherapy, State Key Laboratory of Photocatalysis on Energy and Environment, College of Chemistry, Fuzhou University, Fuzhou 305108, China

^2^Key Laboratory of Laboratory Medicine, Ministry of Education of China, and Zhejiang Provincial Key Laboratory of Medical Genetics, School of Laboratory Medicine and Life Sciences, Wenzhou Medical University, Wenzhou, 325035, China

^3^College of Chemistry and Food Science, Nanchang Normal University, Nanchang, 330032, China

^#^Contributed equally to this work.

*Corresponding authors. E-mail addresses: wuzaisheng@163.com (Z.-S. Wu)

**Table of content**

**S1. Supporting experimental procedures3**

**S1.1 Materials3**

**S1.2 Construction of S-ARGN3**

**S1.3 Determination of the number of Apt-anchor on AuNR surface4**

**S1.4 Gel electrophoresis5**

**S1.5 Atomic force microscope (AFM) imaging5**

**S1.6 The loading of siRNA-Plk1 and stimuli-responsive release5**

**S1.7 Preparation of DS-ARGN5**

**S1.8 Cell culture6**

**S1.9 Confocal fluorescence imaging6**

**S1.10 Flow cytometry analysis7**

**S1.11 Dox loading and in vitro release7**

**S1.12 Evaluation of PLK1 expression level by western blot assay8**

**S1.13 The qPCR assay8**

**S1.14 Establishment of tumor-bearing animal model8**

**S1.15 Evaluation of photothermal performance and in vivo thermal imaging8**

**S1.16 Cell viability assay based on Calcein AM/PI staining9**

**S1.17 Cytotoxicity assay10**

**S1.18 In vivo and in vitro fluorescence imaging10**

**S1.19 Evaluation of anti-tumor activity in vivo10**

**S1.20 Histopathological analysis by hematoxylin and eosin (H&E) staining10**

**S2. Supporting table12**

**Table S1. DNA sequences designed in this work 12**

**S3. Supporting figures13**

**Scheme S1. Schematic diagram of possible local structure of S-ARGN13**

**Scheme S2. Schematic representation of structural reconfiguration of ARGN14**

**Figure S1. The nPAGE analysis (15%) of RCA-p15**

**Figure S2. Optimization of the ratio of DNA components16**

**Figure S3. Verification of the release of D-siRNA-Plk117**

**Figure S4. Characterization of AuNR19**

**Figure S5. Characterization of S-ARGN and counterparts by transmission electron microscopy (TEM)20**

**Figure S6. Characterization of C-ARGN, which was essentially ARGN cross-linked with a doubly-thiolated DNA strand crosslinker (D-crosslinker).21**

**Figure S7. Determination of the number of Apt-anchor on A-AuNR22**

**Figure S8. Determination of RCA-p loading capacity23**

**Figure S9. The siRNA loading capability of S-ARGN25**

**Figure S10. The nuclease degradation-resistance of S-ARGN26**

**Figure S11. Exploration of the long-term stability of S-ARGN 28**

**Figure S12. The internalization efficiency of S-ARGN into L02 cells29**

**Figure S13. The release of siRNA-Plk1 from S-ARGN in response to intracellular miR-2130**

**Figure S14. Evaluation of the long-time stability of S-ARGN within living cells32**

**Figure S15. The standard linear calibration curve between Dox fluorescence intensity and its concentration34**

**Figure S16. Estimation of Dox loading capacity of S-ARGN and release performance of Dox35**

**Figure S17. Evaluation of aptamer-dependent tumor cell-targeted Dox delivery of S-ARGN36**

**Figure S18. Synergistic effects of multimodal combination cancer therapy37**

**Figure S19. Cytotoxicity of S-ARGN-D combined with 808 nm laser irradiation to cancerous and normal cells38**

**Figure S20. Histological analysis of main organs of HeLa tumor-bearing nude mice by H&E stained sections39**

**S4. Supporting references 41**

**S1.Supporting experimental procedures**

**S1.1 Materials**

The oligonucleotides designed in this study were synthesized by Biologycal Gene Technology Co., Ltd (Fuzhou, China). The label-free oligonucleotides were purified by denaturing PAGE, while the chemically-modified oligonucleotides were purified by high-performance liquid chromatography (HPLC). The base sequences of all oligonucleotides are shown in **Table S1**. 1×TEBuffer (pH 7.4, 10 mM Tris-HCl, 1 mM EDTA) was used for the preparation of DNA stock solutions. PEG-modified gold nanorods (AuNRs) were purchased from So-Fe Biomedicine Technology Co., Ltd. (Shanghai, China). Exonuclease I supplied with 10× Exonuclease I Reaction Buffer and Exonuclease III supplied with 10× NEBuffer 1 were purchased from New England Biolabs Inc (Beijing, China). Anti-PLK1 mouse monoclonal antibody, HRP-conjugated goat anti-mouse IgG and doxorubicin hydrochloride (Dox) were purchased from Sangon Biotech. Co., Ltd. (Shanghai, China). Calcein/PI Cell Viability/Cytotoxicity Assay Kit, β-Actin Rabbit Monoclonal Antibody and HRP-labeled Goat Anti-Rabbit IgG were supplied by Beyotime Biotech. Co., Ltd. (Shanghai, China). T4 DNA Ligase, Phi29 MAX DNA Polymerase, ExFect2000 Transfection Reagent, RNA isolater Total RNA Extraction Reagent, HiScript II Q RT SuperMix for qPCR, ChamQ Universal SYBR qPCR Master Mix, miRNA 1st Strand cDNA Synthesis Kit (by stem-loop), miRNA Universal SYBR qPCR Master Mix Kit, BCA Protein Quantification Kit, high-sensitivity ECL chemiluminescence detection kit and CCK-8 Cell Counting Kit were purchased from Vazyme Biotech Co., Ltd. (Nanjing, China). Penicillin-streptomycin (Genview, USA), Roswell Park Memorial Institute (RPMI)-1640 medium (HyClone, USA), Dulbecco’s Modified Eagle Medium (DMEM, HyClone, China), Fetal Bovine Serum (Genview, USA), Hoechst 33342, SYBR Green I, RIPA Lysis buffer, Phenylmethanesulfonyl fluoride (PMSF) and PVDF blotting membrane (GE Healthcare life science) were supplied by Dingguo Changsheng Biotech. Co., Ltd. (Beijing, China). Other reagents used were of analytical grade. Ultrapure water (>18.25 MΩ) purified by Kerton lab MINI water purification system (UK) was used for the preparation of all aqueous solutions.

**S1.2 Construction of S-ARGN**

***Preparation of Circle*:**

Equal volumes (1 μL) of Circle-1 (10 μM), Circle-2 (10 μM), Template-1 (10 μM) and Template-2 (10 μM) were mixed with 2 μL of 10× Ligase buffer. After adjusting the volume to 19 μL with ddH_2_O, 1 μL of T4 DNA Ligase (400 U/μL) was added and mixed. The resulting solution was stored at 16 ℃ for 12 h and then DNA Ligase was inactivated at 65 ℃ for 10 min. The cyclized product was named Circle.

***Preparation of RCA-p*:**

A 4-μL aliquot of 10× phi 29 DNA polymerase buffer, 1 μL of dNTPs (10 mM), 0.5 μL of phi29 DNA polymerase (10 U/μL) and 14.5 μL of ddH_2_O were added into 20 μL of the Circle pre-prepared above. The resulting solution was mixed well and stored for 30 min at 30 °C. Then, the temperature was increased to 65 °C and incubated for 10 min to inactivate the phi29 DNA polymerase and terminate the rolling circle amplification reaction. Consequently, a crude RCA product (RCA-p) (final volume, 40 μL, called V_f_) was obtained.

Subsequently, the crude RCA product was purified by the followed procedure. Firstly, a solution for the purification (p-Solution) was prepared by mixing phenol, chloroform and isoamyl alcohol at a volume ratio of 25:24:1. The crude RCA-p was mixed with an equal volume of p-Solution and gently shook for 20 s, followed by centrifugation (12000 rpm) for 5 min at room temperature and removal of the supernatant. Then, 4 μL (10% of V_f_) of sodium acetate (3 M), 100 μL (2.5 times V_f_) of absolute ethanol and 1 μL of Glycogen (5 mg/mL) were added, followed by storage at -80 ℃ for 2 h. After centrifugation (4 ℃, 16000 rpm) for 30 min, the supernatant was discarded, and 250 μL of 70% ethanol was used for washing the precipitates two times by centrifugation. Subsequently, the ethanol was allowed to evaporate and ddH_2_O was added to dissolve the purified RCA-p. The concentration was determined by a Q5000 UV/vis spectrophotometer (Quawell Technology, Inc., USA). The concentration of basic structural units (RCA unit) was defined as the concentration of RCA-p. Moreover, the concentration of RCA-p was adjusted to 10 μM and stored at 4 °C in the dark before use.

***Preparation of siRNA-Plk1:***

D-Plk1-antisense (10 μM) and D-Plk1-sense (10 μM) were added to a certain volume of PBS (136.89 mM NaCl; 2.67 mM KCl; 8.1 mM Na_2_HPO_4_ and 1.76 mM KH_2_PO_4_, pH 7.4) and the final concentration of each strand was 1 μM. After annealing at 90 ℃ for 5 min, the resulting mixture was slowly cooled down to 25 ℃ and stored at 4 ℃, generating D-siRNA-Plk1. During the evaluation of RNA interference therapeutics, including the western blot analysis, qPCR analysis, Cell viability assay based on Calcein AM/PI staining, cytotoxicity analysis and tumor treatment *in vivo*, D-Plk1-antisense and D-Plk1-sense were substituted with corresponding RNA sequences, Plk1-antisense and Plk1-sense, respectively, to exert RNA interference therapy. The corresponding product was named siRNA-Plk1.

***Preparation of A-AuNR***

Apt-anchor, which is labeled with a 5’-end sulfhydryl group, was used to functionalize gold nanorods (AuNRs) according to the reported method[1] with slight modification. Specifically, 600 μL of AuNRs (0.1 nM) was centrifuged for 5 min at the speed of 8000 rpm, the supernatant was removed and 500 μL of 0.01% tween-20 was used to resuspend the pellet. The same process was repeated three times, resulting in Solution A. The sulfhydryl-labeled Apt-anchor (8 μL, 100 μM) and Tris (2-carboxyethyl) phosphine (TCEP, 4 μL, 10 mM) were mixed uniformly and stored for 1 h at room temperature, resulting Solution B. Then, Solution B was mixed with Solution B, followed by addition of 56.9 μL of sodium citrate (1 M) and incubation for 1 h. Afterwards, the resulting solution was centrifuged, and the pellet was resuspended in 500 μL of PBS. The centrifugation/resuspension step was repeated two times, obtaining Aptamer-anchor modified gold nanorod solution (A-AuNR, 500 μ L) that was stored at 4 °C in the dark before usage.

***Preparation of S-ARGN***

The RCA-p (1.73 μL, 10 μM) was mixed with A-AuNR (200 μL, 120 pM). The resulting solution was annealed at 90 ℃for 5 min and slowly cooled down to 25 ℃, generating ARGN. Then, 35 μL of siRNA-Plk1 (1 μM) prepared above was added and stored for 8 h at room temperature. After centrifugation at 8000 rpm for 5 min, the supernatant was discarded and the pellet was resuspended in 500 μL of PBS. The centrifugation/resuspension step was repeated two times, and the pellet was finally resuspend in 200 μL of PBS, obtaining S-ARGN (120 pM) whose concentration was estimated from the concentration of AuNRs.

Two control samples are described as follows: ARGN was used as the control without siRNA-Plk1. For S-RRGN control, the preparation procedure is the same as S-ARGN except that Apt-anchor was substituted with R-anchor.

**S1.3 Determination of the number of Apt-anchor on AuNR surface (Figure S7)**

The Apt-anchor attached on the surface of gold nanorod was quantified according to the reported method.[1, 2] In short, Cy5-labeled Apt-anchor (Cy5-Apt-anchor) was modified to the surface of AuNRs by the method of “*Preparation of A-AuNR*”. Then, 600 μL of the resulting product solution was centrifuged (8000 rpm) and the supernatant was discarded. After 200 μL of mercaptoethanol (20 mM) was used to resuspend the pellet and allowed to react at 37 °C for 5 h to replace fully Cy5-Apt-anchor. Subsequently, the AuNR precipitate was removed by centrifuge (8000 rpm), and the Cy5 fluorescence intensity of the supernatant was detected by a fluorescence spectrometer (Hitachi Ltd, Japan). The excitation wavelength was set at 585 nm, the voltage was 800 V and the excitation/emission slit widths were 10 nm.

**S1.4 Gel electrophoresis (Figure 1A，Figure 3B and 3C，Figure S1, Figure S2, Figure S3, and Figure S8)**

A gel electrophoresis instrument (Bio-Red, USA) was used to perform the natural polyacrylamide gel electrophoresis (nPAGE, 15%). To prepare the sample, an 8-μL aliquot of analyte solution was mixed with 2 μL of 6× SYBR Green I and 2 μL of 6×loading buffer. The resulting solution was loaded into the gel wells, followed by nPAGE analysis during which 0.5×TBE buffer (4.5 mM Tris-HCl, 4.5 mM boric acid and 0.1 mM EDTA, pH=7.9) was engaged as the working buffer. Gel imaging was by an imaging system ChemiDoc XRS (Bio-Red, USA).

**S1.5** **Atomic force microscope (AFM) imaging (Figure 1B)**

A 10-μL aliquot of analyte sample was dropped on the surface of freshly-cleaved mica and incubated for 30 min. Then, the mica surface was rinsed four times with ddH_2_O and dried by a pure nitrogen stream. The AFM imaging was performed by a MultiMode 8 atomic force microscope (Bruker, Germany).

**S1.6 The loading of siRNA-Plk1 and stimuli-responsive release (Figure 3B and 3C).**

***Figure 3B:***

The RCA-p (0.87 μL, 10 μM) or Structural unit of RCA product (SUR) (0.87 μL, 10 μM) was mixed with A-AuNR (50 μL, 240 pM). The resulting solution was annealed at 90 ℃ for 5 min and slowly cooled down to 25 ℃. Then, 20 μL of siRNA-Plk1 (1 μM) was added and incubated at room temperature for 8 h. After centrifugation at 8000 rpm for 5 min, the supernatant was collected for gel electrophoresis analysis according to the section of S1.4.

***Figure 3 C:***

***For lane 1 and Lane 3:*** The RCA-p (0.87 μL, 10 μM) or SUR (0.87 μL, 10 μM) was mixed with A-AuNR (100 μL, 120 pM). The resulting solution was annealed at 90 ℃ for 5 min and slowly cooled down to 25 ℃. Then, 17.5 μL of D-siRNA-Plk1 (1 μM) was added and incubated at room temperature for 8 h. After centrifugation at 8000 rpm for 5 min, the supernatant was discarded, and the pellet was resuspended in 250 μL of PBS. The centrifugation/resuspension process was repeated two times, and the pellet was finally resuspend in 50 μL of PBS, obtaining S-ARGN (240 pM) or S-AUG (240 pM).

***For lane 2 and Lane 4:*** S-ARGN (50 μL, 240 pM) or S-AUN (50 μL, 240 pM) was mixed with D-miRNA-21 (2 μL, 10 μM) and incubated at room temperature for 4 h.

***For Lane 5****:* D-Plk1-antisense (1.75 μL, 10 μM) and D-Plk1-sense (1.75 μL, 10 μM) were added to PBS (14 μL) and annealed at 90 ℃ for 5 min. The resulting mixture was slowly cooled down to 25 ℃, generating D-siRNA-Plk1.

**S1.7 Preparation of** **DS-ARGN**

Briefly, 2630 μL of S-ARGN (120 pM) was mixed with 50 μL of Dox (50 μM) and stored at room temperature for 12 h. After centrifugation at 8000 rpm for 5 min, the supernatant was removed and the precipitate was resuspended in 1 mL of PBS. The centrifugation was repeated again, and the precipitate was finally resuspended in 200 μL of PBS, generating DS-ARGN in which the equivalent concentration of Dox was 2.5 μM.

**S1.8 Cell culture**

Human breast adenocarcinoma MCF-7 cell lines and human cervical cancer HeLa cell lines were employed as positive cells, which were cultured in DMEM medium. RPMI-1640 medium was used for culturing normal human hepatocytes (L02) as negative cells. Both the media were supplemented with 10% fetal bovine serum, 100 U/mL penicillin and 100 μg/mL streptomycin. The three kinds of cells were all cultured at 37 °C for about 24 h in a humidified incubator maintained at 5% CO_2_ atmosphere before use.

**S1.9** **Confocal fluorescence imaging**

***Exploration of the targeting ability of S-ARGN (Figure 2A and Figure S12A):***

MCF-7 and L02 cells were separately cultured in a 12-well plate at a density of 1.0×10^5^ cells/well. After removal of the medium, the cells were washed with PBS three times. Cy5-labeled S-ARGN was prepared as follows: Apt-anchor was firstly used to modify gold rod. Then, following the procedure of "Preparation of S-ARGN", Cy5-labeled S-ARGN was assembled where Cy5-siRNA-Plk1 was used instead of label-free siRNA-Plk1. The Cy5-labeled S-ARGN (200 μL, 120 pM) was mixed well with 300 μL of DMED or RPMI-1640 medium and then incubated with cells (one well) for 4 h. The final concentration of Cy5-labeled S-ARGN was 48 pM. After the medium was removed and the cells were washed three times with PBS, the resulting cells were fixed with 4% paraformaldehyde for 15 min and washed three times with 500 µL of PBS. Subsequently, the nuclei were stained with Hoechst 33342 (500 µL, 10 µg/mL) at 37 °C for 15 min. The residual reagents were removed, and the cells were washed with 500 µL of PBS. Finally, confocal fluorescence imaging was performed by Leica SP8 laser scanning confocal microscope (Leica, Germany). The excitation wavelengths of 405 nm and 638 nm were used to excite Hoechst and Cy5, respectively.

***Controlled release of siRNA-Plk1 (Figure S13A):***

β-Estradiol (E_2_) was used to reduce the expression level of miR-21 in MCF-7 cells. Briefly, MCF-7 cells were cultured in DMEM medium containing 10 nM of E_2_ for 24 h, and the treated cells were called MCF-7-E_2_. The density of MCF-7 cells or MCF-7-E_2_ seeded in a 12-well plate was 1.0×10^5^ cells/well. The Cy5-labeled S-ARGN (200 μL, 120 pM) was mixed with 300 μL of DMED medium uniformly and incubated with the cells for 4 h. Subsequently, the medium containing residual Cy5-labeled S-ARGN was discarded, and the cells were washed three times with 500 μL of PBS. After cell fixation and nuclear staining treatment described above, the confocal fluorescence imaging was performed.

***Evaluation of the delivery efficiency of siRNA-Plk1 (Figure 3D)***:

HeLa cells were cultured in a 12-well plate at a density of 1.0×10^5^ cells/well. After the culture medium was discarded, the cells were washed three times with PBS. **For S-ARGN group**, Cy5-labeled S-ARGN (200 μL, 120 pM) was mixed with 300 μL of DMED medium and incubated with cells for 4 h. The remaining operations were the same as “***Exploration of targeting ability of S-ARGN***”. The **Naked siRNA-Plk1 group** was prepared according to the same procedure but the equal amount of siRNA-Plk1 was instead used. **For S-AFR group:** Apt-anchor (0.5 μL, 10 μM) and RCA-p (0.4 μL, 10 μM) were mixed with 9.1 μL of PBS and annealed at 90 ℃ for 5 min, immediately followed by cooling gradually to room temperature. Subsequently, 6.8 μL of D-siRNA-Plk1 (1 μM) was added and incubated at room temperature for 4 h, forming the S-AFR. After 183.2 μL of PBS and 300 μL of DMED medium were added and mixed well, the resulting solution was used to culture HeLa cells for 4 h. **For Lipo-siRNA-Plk1 group**, all experiments were preformed according to the instructions of ExFect2000 Transfection Reagent. Specifically, cells were pre-cultured in a 12-well plate, and the medium was removed when the cell confluence reached 60%. The cells were washed three times with 500 μL of PBS, followed by addition of 400 μL of medium. ExFect2000 transfection reagent (3 μL) was added into 50 μL of opti-MEM, mixed gently and stored for 5 min. Then, 6.8 μL of prepared siRNA-Plk1 (1 μM, labeled with Cy5) was added to 50 μL of opti-MEM, mixed gently and stored for 5 min. Subsequently, the diluted siRNA-Plk1 and diluted ExFect2000 transfection reagent were mixed uniformly and stored for 10 min. The resulting mixture was added into the above-mentioned cultured cells, shaken gently and cultured in an incubator for 4 h. Afterwards, the culture solution was replaced with fresh culture medium, followed by incubation in an incubator overnight. After cell fixation and nuclear staining, the confocal fluorescence imaging was performed. The equivalent concentration of siRNA-Plk1 was used in each experiment group, and its final concentration was 13.3 nM.

***Interrogation of cell-targeted delivery of Dox (Figure S17A)***:

200 μL of DS-ARGN (the equivalent concentration of Dox, 2.5 μM) was prepared according to the section of **S1.7** “Preparation of DS-ARGN” and was mixed with 300 μL of DMEM medium, and the final concentration of Dox was 1 μM. The resulting medium was used to incubate with the cells (pre-cultured in the 12-well plate) for 4 h. After discarding the medium, the cells were washed three times with 500 μL of PBS to remove the remaining material. Confocal fluorescence imaging was performed after cell fixation and nuclear staining. The excitation wavelengths of Hoechst and Dox were set at 405 nm and 488 nm, respectively.

**S1.10 Flow cytometry analysis**

***Specific cell uptake analysis (Figure 2C and Figure S12B)***: The cells were seeded in a 12-well plate and pre-cultured for 24 h. After removing the medium, the cells were washed three times with 500 μL of PBS. Then, Cy5-labeled S-ARGN (200 μL, 120 pM) was mixed with 300 μL of DMED or RPMI-1640 medium and incubated with the cells for 4 h. Subsequently, the used medium was discarded and the cells were washed three times with 500 μL of PBS. Finally, the cells were digested with 0.25% trypsin, centrifuged at 1400 rpm for 5 min and resuspended in 500 μL of PBS. Flow cytometry analysis was conducted on a CytoFLEX flow cytometry (Beckman Coulter, Inc., America), and the data were analyzed with the help of FlowJo software.

***Evaluation of cell-targeted delivery of Dox (******Figure S17B)***: The treatment of cells with DS-ARGN was performed according to the same procedure as described in the section of **S1.9** “***Interrogation of cell-targeted delivery of Dox (Figure S17A)****”.* Subsequently, the treated cells were digested with 0.25% trypsin, collected by centrifugation (1400 rpm, 5 min) and resuspended in 500 μL of PBS, followed by flow cytometry analysis.

**S1.11 Dox loading and *in vitro* release**

***Dox loading into S-ARGN (Figure S16A and S15B)***: Dox (46 μL, 1 μM) was mixed with different volumes of S-ARGN at a specific concentration and the total volume was supplemented with PBS to 200 μL, followed by incubation at room temperature for 12 h. The final concentration of Dox in each sample was equivalent to 230 nM and remained unchanged, while the final concentration of S-ARGN was 2 pM, 8 pM, 20 pM, 80 pM, 125 pM or 300 pM. The fluorescence spectrum of Dox ranging from 500 nm to 700 nm was collected at the excitation wavelength of 470 nm on a fluorescence spectrometer (Hitachi Ltd., Japan). The photomultiplier tube voltage was set as 700 V, while the excitation and emission slits were 5 nm.

***In vitro release of Dox (Figure S16C)*:** The preparation of DS-ARGN was the same as the description of **S1.7** “Preparation of DS-ARGN” except the final equivalent concentration of Dox was adjusted to 2 μM (C_0_) and the total volume was 400 μL. The DS-ARGN was incubated in a shaker at 200 rpm and 37 ℃ for 12 h during which a 808 nm NIR laser irradiation (0.5W/cm^2^) was performed for 5 min every 30 min. Subsequently, the sample was centrifuged (8000 rpm, 5 min), and the supernatant was collected. The fluorescence intensity of Dox was measured on a fluorescence spectrometer and was converted to the molar concentration of Dox (C_a_) in the supernatant by interpolation from a standard linear calibration curve (**Figure S15**). The release efficiency (RE) of Dox was calculated by the following formula: RE= C_a_/ C_0_×100%.

**S1.12 Evaluation of PLK1 expression level by western blot assay (Figure 3F)**

S-ARGN (148 μL, 1.2 nM) was diluted by supplementing with 352 μL of PBS and added into 500 μL of DMEM. After mixing thoroughly, the equivalent concentration of siRNA-Plk1 was 50 nM. Then, S-ARGN-containing medium was added to the pre-cultured HeLa cells (8.0×10^5^) in a 6-well plate and incubated for 24 h. The resulting cells were collected to extract the total protein, and the protein concentration was determined by the BCA Protein Quantification Kit. Lipo-siRNA-Plk1 group was prepared with the help of ExFect2000 transfection reagent to deliver siRNA-Plk1 into the cells based on the instructions. The equivalent concentration (50 nM) of siRNA-Plk1 was used in all treatment groups. 50 μg of total protein extracted from the cells of different treatment groups was separated by SDS-PAGE (10%) and transferred to PVDF blotting membrane. After blocking the non-specific binding sites by incubation with 5% skim milk, the PVDF membrane was incubated sequentially with the primary antibody (Anti-PLK1 mouse monoclonal antibody or β-Actin Rabbit Monoclonal Antibody) and secondary antibody (HRP-conjugated goat anti-mouse or IgG HRP-labeled Goat Anti-Rabbit IgG). Subsequently, 500 μL of ECL working solution was added and incubated for 2 min based on the instruction of high-sensitivity ECL chemiluminescence detection kit. The band of PLK1 was visualized by ChemiDoc XRS+ (BIO-RAD, USA) where β-actin served as the quantitative internal reference.

**S1.13** **The qPCR assay (Figure 3E and Figure S13B)**

The treatment of the cells was performed according to the same procedure as the section of **S1.12**. RNA isolater Total RNA Extraction Reagent was used to obtain total RNA, 1 μg of which was used for the preparation of cDNA (20 μL) based on the instruction of HiScript II Q RT SuperMix for qPCR. The mRNA level of PLK1was analyzed by ChamQ Universal SYBR qPCR Master Mix and evaluated by the 2^-(∆∆Ct)^ method. Endogenous U6 small RNA was used as the internal reference.

**S1.14** **Establishment of tumor-bearing animal model**

BALB/c nude mice (4-6 weeks old, female, 20-25 g) used for *in vivo* studies were purchased from Wushi Experimental Animal Trading Co., Ltd. (Fuzhou, China). The management and use of experimental animals comply with the regulations of the Experimental Animal Ethics Committee of Fuzhou University and the Laboratory Animal Management Committee of Fuzhou University (approval number: SYXK-2019-0007). HeLa cells (100 μL, 8.0×10^6^) were subcutaneously inoculated into the right flanks of nude mice to establish the tumor-bearing nude mouse model. The tumor volume was calculated according to the formula (a×b ^2^/2), where a and b represent the length and width of the tumor, respectively. When the tumor volume reached 100 mm^3^, the *in vivo* anticancer treatment was performed.

**S1.15** **Evaluation of photothermal performance and *in vivo* thermal imaging**

**Evaluation of photothermal performance of S-ARGN (Figure 4A-4E)**

For **Figure 4A** and **4B**: S-ARGN (850 μL) was centrifuged at 8000 rpm for 5 min. After removing the supernatant, the precipitate was dissolved in PBS, and the concentration was adjusted to 2 mg/mL. Subsequently, 50 μL of resulting solution was used to determine the laser irradiation-dependent temperature change curve. During irradiating the sample with 808 nm laser at a power intensity of 0.5 W/cm^2^, the temperature was real-time monitored on an infrared thermal imaging camera (FOTRIC 225s, USA). The control samples were prepared to contain the same concentration (2 mg/L) of bare AuNR or AuNR modified with different molecules. The laser irradiation-induced temperature change was monitored under identical conditions.

For **Figure 4C**: Different concentrations (0.10, 0.12, 0.20, 1.00 and 2.00 mg/mL) of S-ARGN (50 μL) were separately prepared and irradiated by 808 nm laser at a power intensity of at 0.5W/cm^2^ for 4 min, during which the temperature change was monitored in real time. The data was processed by AnalyzIR software.

For **Figure 4D**: S-ARGN (2 mg/mL, 50 μL) was irradiated by 808 nm laser with various laser intensities (0.25, 0.38, 0.44 or 0.50 W/cm^2^) for 4 min, during which the temperature change was monitored in real time. The data were processed by AnalyzIR software.

For **Figure 4E**: S-ARGN (2 mg/mL, 50 μL) was irradiated with 808 nm laser (0.50 W/cm^2^) for 4 min, following by turning off the infrared light for 5 min. The laser irradiation was repeated four times in a cyclical fashion. During this process, real-time temperature monitoring was performed. Infrared thermal imaging and temperature monitoring were performed on an infrared thermal imaging camera (FOTRIC 225s, USA), and the data were processed by AnalyzIR software.

***In vivo* thermal imaging (Figure 5D):**

***For S-ARGN+ Laser group:*** The S-ARGN (100 μL, 2.4 nM) was injected into tumor-bearing nude mice via the tail vein and circulated for 2 h. After the tumor site was continuously irradiated with 808 nm laser (0.5 W/cm^2^) for 1 min, the infrared thermal imaging was immediately performed. ***For S-ARGN group:*** the administration of S-ARGN and infrared thermal imaging were performed under identical conditions but not exposed to 808 nm laser irradiation. ***For the PBS group***: the experiments were conducted following the same procedure as S-ARGN+ Laser group except that S-ARGN was substituted with PBS. The infrared thermal imaging and temperature monitoring were performed on an infrared thermal imaging camera (FOTRIC 225s, USA), and the data were processed by AnalyzIR software.

**S1.16 Cell viability assay based on Calcein AM/PI staining** **(Figure 4F)**

Calcein AM (Calcein Acetoxymethyl Ester) and PI (propidium iodide) were sued to stain the cells of interest because they can stain live and dead cells, respectively. **For No Laser group**: 158 μL of ARGN (1.2 nM), S-ARGN (1.2 nM; the equivalent concentration of siRNA-Plk1 was 0.1 μM) and DS-ARGN (1.2 nM; the equivalent concentrations of Dox and siRNA-Plk1were 0.6 μM and 0.1 μM, respectively.) were separately mixed with 342 μL of DMEM medium and incubated with HeLa cells (0.5×10^5^) for 13 h. Then, the nanoformulation-containing culture medium was discarded. **For Laser group:** 158 μL of ARGN (1.2 nM), S-ARGN (1.2 nM; the equivalent concentration of siRNA-Plk1 was 0.1 μM) and DS-ARGN (1.2 nM; the equivalent concentrations of Dox and siRNA-Plk1were 0.6 μM and 0.1 μM, respectively.) were separately mixed with 342 μL of DMEM medium and incubated with the cells (0.5×10^5^) for 12 h. Then, the nanoformulation-containing culture medium was replaced with fresh medium and an 808 nm laser light (0.5 W/cm^2^) was used to irradiate the cells for 5 min. Afterwards, the cells were continued to be cultured for 1 h, followed by fully removing the medium. The cells obtained from No Laser group and Laser group were separately incubated with 250 μL of Calcein AM/PI detection working solution for 30 min at 37 ℃. Finally, a Leica SP8 laser scanning confocal microscope (Leica, Germany) was used for fluorescence imaging, and Calcein AM and PI were excited with wavelengths of 488 nm and 552 nm, respectively.

**S1.17 Cytotoxicity assay (Figure S18A and Figure S19)**

Pre-cultured cells (MCF-7, HeLa or L02) (0.5×10^5^) in a 96-well plate were incubated with 100 μL of medium containing a given concentration of ARGN, D-ARGN, S-ARGN or DS-ARGN for 24 h. **For the laser irradiation treatment group**, the medium containing nanoformulations was used to culture the cells for 24 h and then replaced with fresh medium. The resulting cells were exposed to an 808 nm laser at the power density of 0.5 W/cm^2^ for 5 min. Afterwards, the cells were washed three times with PBS and incubated with fresh culture medium containing 10% CCK-8 (100 μL/well) for 2 h. A microplate reader (TECAN Infinite F200) was used for the detection of the absorbance at 450 nm.

To evaluate the intrinsic cytotoxicity of S-ARGN (not exposed to laser irradiation) and influence of laser irradiation on cell viability (**Figure S19**), all the experiments were performed according to the same procedure as above, except that only S-ARGN at the given concentration was involved. The cell viability of native cells (not exposed to S-ARGN) is defined as 100%.

**S1.18 *In vivo* and *in vitro* fluorescence imaging (Figure 5A, 5B and 5C)**

S-ARGN and S-RRGN (100 μL, 120 pM) labeled with Cy5 (i.e., Cy5-Plk1-antisense was instead used during the assembly) were separately injected into two groups of tumor-bearing nude mice through the tail vein. *In vivo* fluorescence imaging was performed by IVIS Lumina LT Series III Imaging Spectrum System (PerkinElmer, USA) at different time points (1 h, 2 h and 4 h) post-administration. Moreover, finally, the mice were sacrificed by cervical dislocation, and their tumors and major organs were collected for *in vitro* fluorescence imaging. The IVIS Living Imaging 3.0 software was used to analyze the fluorescence images.

**S1.19 Evaluation of anti-tumor activity *in vivo* (Figures 6A, 6B and 6C)**

The DS-ARGN-based treatment was performed when the tumor volume of HeLa-tumor bearing nude mice reached 100 mm^3^. The tumor-bearing nude mice were randomly divided into five groups each with five mice, and they were administrated with 100 μL of PBS, Dox, D-ARGN or DS-ARGN via single tail vein injection. Except for PBS group, the equivalent dose of Dox was 40 μg/kg, while the siRNA-Plk1 dose was 0.25 mg/kg for the groups of DS-ARGN and DS-ARGN+Laser. The intravenously injection was performed every 3 days for 18 consecutive days. For DS-ARGN+Laser group, the photothermal therapy was conducted at 4 h each post-administration. The tumor site was irradiated once every 30 min with an 808 nm laser with a power density of 0.5 W/cm^2^ for 1 min, and the laser irradiation was performed 10 times (corresponding to the total laser exposure time of 10 min after each administration of drug nano-formulation). The tumor volume and the body weight of nude mice were recorded at 24 h post-administration. The tumor-bearing nude mice were finally sacrificed after 18 days of treatment. The tumors and major organs, including heart, liver, spleen, lung and kidney, were harvested for *ex vivo* photographing and histological analysis.

**S1.20 Histopathological analysis by hematoxylin and eosin (H&E) staining (Figures 6D and S20)**

The tumors and organs, including heart, liver, spleen, lung, and kidney, were fixed by incubation in 4% cold paraformaldehyde solution for 48 h. Then, the tissues were embedded into paraffin, sectioned into slices with the thickness of 5 μm, and stained with hematoxylin and eosin (H&E). Histological analysis was carried out by imaging on a Primo Star Upright Biological Microscope equipped with AxioCam ERc5s microscope camera (Carl Zeiss, Germany).

**S2. Supporting table**

**Table S1**. DNA sequences designed in this work^[a]^.

| **Names** | **Sequences** (from 5' to 3') | |
| --- | --- | --- |
| Circle-1 | | **P**-CTGTC CATCA TAGCT TATCA GACTG ATGTT GAAAC ATAAA AACTA CCTGT |
| Circle-2 | | **P**-CCTCA CCAAC TAGCT TATCA GACTG ATGTT GAAAC ATAAA ACAAC CACTC |
| Template-1 | | AGCTA GTTGG TGAGG ACAGG TAGTT TTTAT |
| Template-2 | | AGCTA TGATG GACAG GAGTG GTTGT TTTAT |
| Structural unit of RCA product  (SUR) | | TTTTA TGTTT CAACA TCAGT CTGAT AAGCT AGTTG GTGAG GACAG GTAGT TTTTA TGTTT CAACA TCAGT CTGAT AAGCT ATGAT GGACA  GGAGT GGTTG |
| Aptamer-anchor (Apt-anchor) | | **SH**-CAACC ACTCC TGTCC ATCAT TTACT ACCTG TCCTC ACCAA CTTTT **GGTGG TGGTG GTTGT GGTGG TGGTG G** |
| Cy5-Apt-anchor | | **SH**-CAACC ACTCC TGTCC ATCAT TTACT ACCTG TCCTC ACCAA CTTTT **GGTGG TGGTG GTTGT GGTGG TGGTG G**-**Cy5** |
| Random-anchor (R-anchor) | | **SH**-CAACC ACTCC TGTCC ATCAT TTACT ACCTG TCCTC ACCAA CTTTT ATATA GCGAG TATGT TGTCA ACTGC C |
| Plk1-antisense | | UAAGG AGGGU GAUCU UCUUC AdTdT |
| Plk1-sense | | UGAAG AAGAU CACCC UCCUU AdTdT GACUG AUGUU GAAAC AUA |
| D-Plk1-antisense | | TAAGG AGGGT GATCT TCTTC ATT |
| D-Plk1-sense | | TGAAG AAGAT CACCC TCCTT ATTGA CTGAT GTTGA AACAT A |
| Cy5-Plk1-antisense | | **Cy5-**TAAGG AGGGT GATCT TCTTC ATT |
| D-miRNA-21 | | TAGCT TATCA GACTG ATGTT GA |
| D-miRNA-221 | | AGCTA CATTG TCTGC TGGGT TTC |
| D-miRNA-211 | | AGGCG AAGGA TGACA AAGGG AA |
| D-miRNA-31 | | AGGCA AGATG CTGGC ATAGC T |
| Plk1-forward | | AGCCT GAGGC CCGAT ACTAC CTAC |
| Plk1-reverse | | ATTAG GAGTC CCACA CAGGG TCTTC |
| U6-forward | | CTCGC TTCGG CAGCA CA |
| U6-reverse | | AACGC TTCAC GAATT TGCGT |
| Doubly-thiolated DNA strand crosslinkers (D-crosslinker) | | HS-TT ATTAT GGCCG GGTAT TAAGA AAAAA AAAAA TT-SH |

^[a]^The segments with the same color represent the base sequences complementary to each other. The boldfaced domain in Apt-anchor denotes the AS1411. The boxed fragments of structural unit of RCA product can perfectly hybridize with miRNA-21.

**S3. Supporting figures**


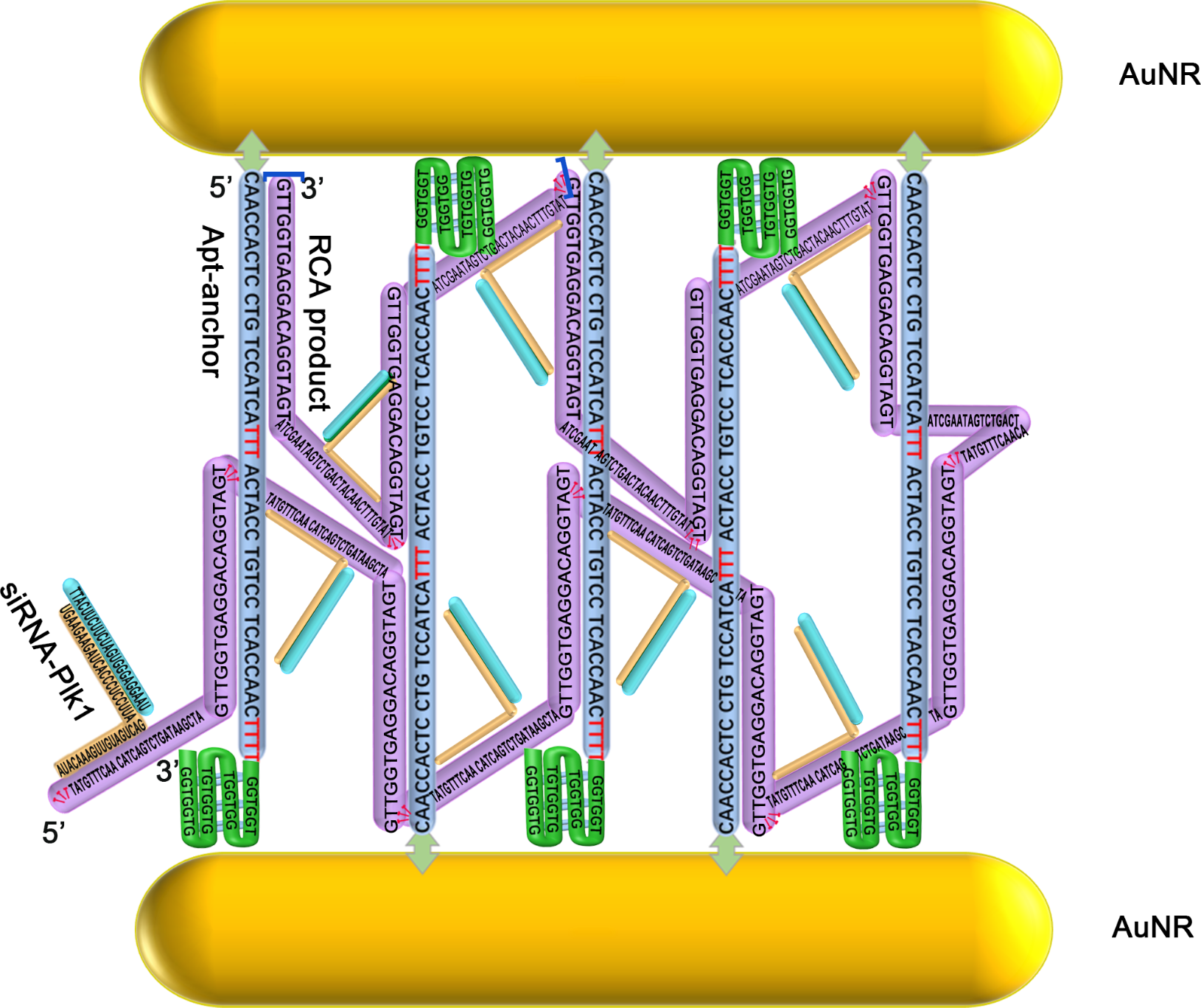


**Scheme S1**. **Schematic diagram of possible local structure of S-ARGN.** Long purple strand represents a partial sequence of RCA-product (RCA-p) consisting of multiple structural units one of which is indicated in the parentheses and contains two binding sites for siRNA-Plk1. The green domain represents aptamer AS1411. The bidirectional arrows indicate the gold-thiol bonds.

**
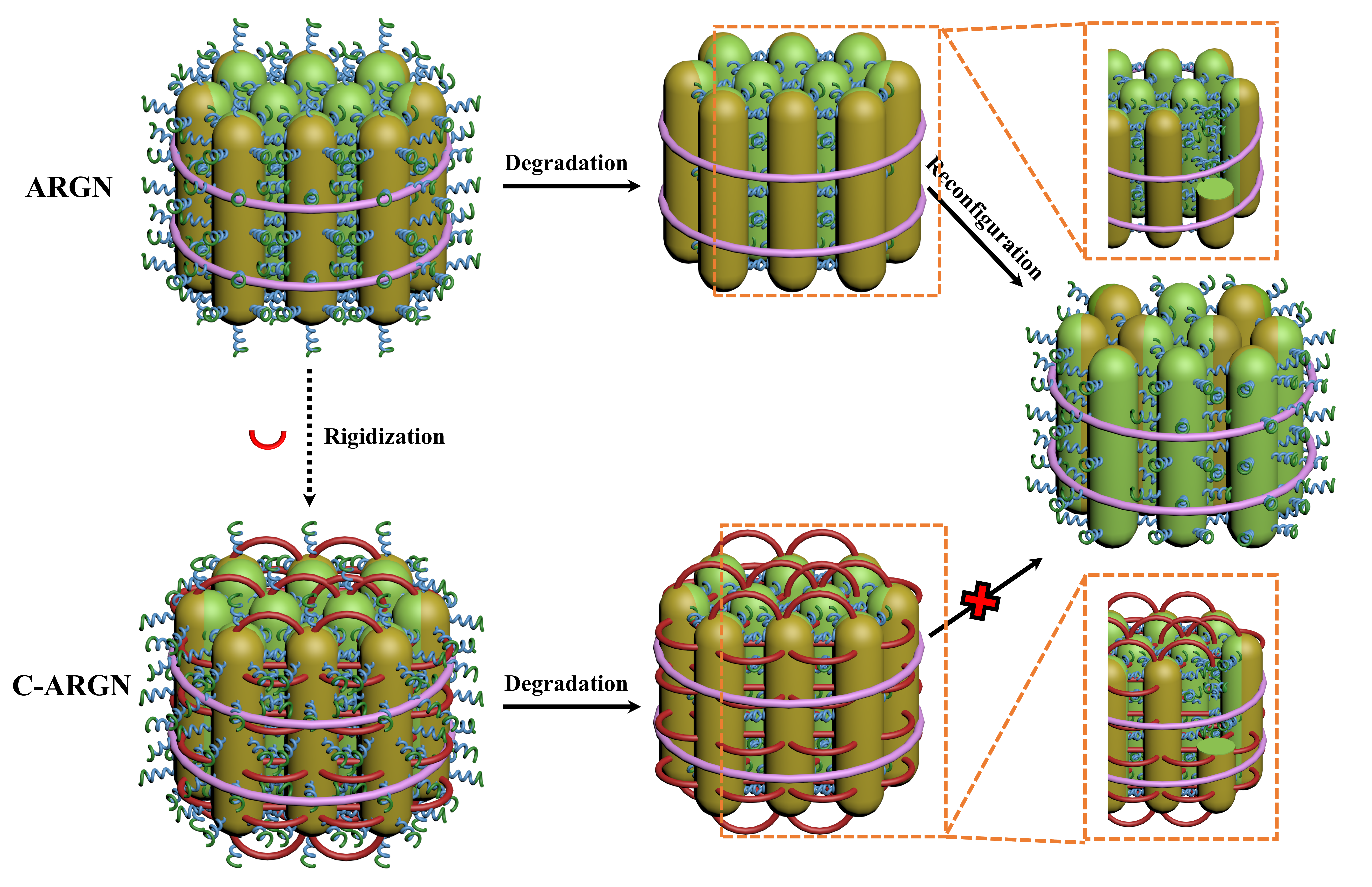
**

**Scheme S2.**  **Schematic representation of structural reconfiguration of ARGN.** ARGN has a high degree of freedom and can reconfigurate its own structure after the enzymatic degradation of nanorod-confined DNA strands on the outside surface, thereby changing the relative position of nanorods and exposing the interior surface-confined DNA strands, including the targeting aptamers. In contrast, the position of each nanorod in ARGN fixed by a crosslinker (called C-ARGN) remains unchanged, and the interior DNA strands are not exposed even if the external DNA strands are completely degraded.


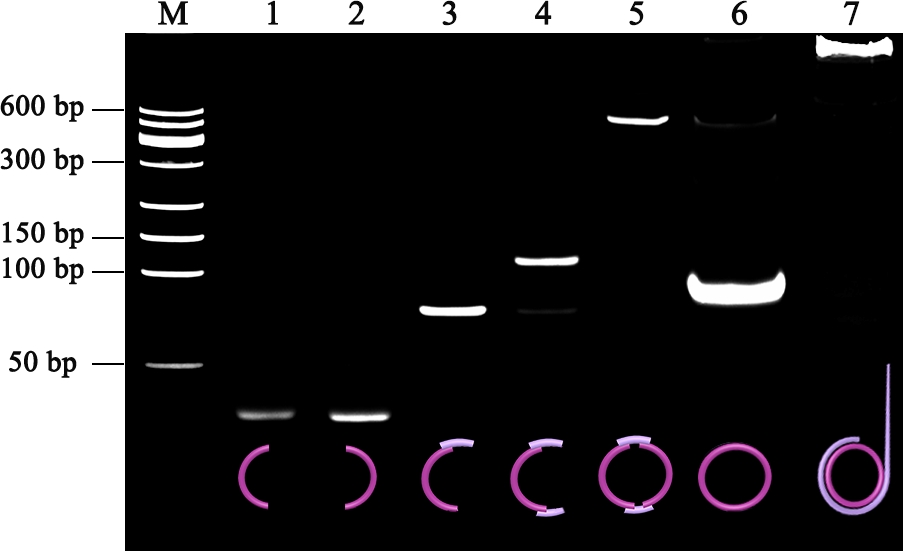


**Figure S1**. **The nPAGE analysis (15%) of RCA-p**. Lane 1: Circle-1; Lane 2: Circle-2; Lane 3: Circle-1+Template-1; Lane 4: Circle-1+Template-1+Template-2; Lane 5: Circle-1+Template-1+Template-2+Circle-2; Lane 6: Purified Circle; Lane 7: RCA product (RCA-p). “M” stands for the low molecular weight DNA Marker. The concentration of each strand in Lanes 1 to 5 was 500 nM. The concentrations of Circle and RCA-p are 5 μM and 10 μM (estimated from the concentration of basic structural unit), respectively.

**Experimental procedure**:

***For Lanes 1-5***: Corresponding DNA strands (each 1 μL, 10 μM) and 2 μL of 10× Ligase buffer were thoroughly mixed, and the total volume was adjusted with ddH_2_O to 20 μL. The samples were annealed at 90 ℃ for 5 min and cooled down to room temperature. The nPAGE analysis was conducted as described in the section of “S1.4 Gel electrophoresis”.

***For Lane 6***: Firstly, 20 μL of Circle prepared according to the section of ‘***Preparation of Circle’*** was mixed with 10× Exonuclease I Reaction Buffer (4 μL), 10× NEBuffer 1 (4 μL), Exonuclease I (0.5 μL, 20,000 units/mL), Exonuclease III (0.5 μL, 100,000 units/mL) and ddH_2_O (11 μL). The resulting solution was stored at 37 ℃ for 1 h and heated to 80 ℃ for 20 min, and the residual product was name Purified Circle. The nPAGE analysis was conducted as described in the section of “***S1.4 Gel electrophoresis***”.

***For Lane 7***：The sample was prepared according to the section of ‘***Preparation of RCA-p’***. The nPAGE analysis was conducted as described in the section of “***S1.4 Gel electrophoresis***”.


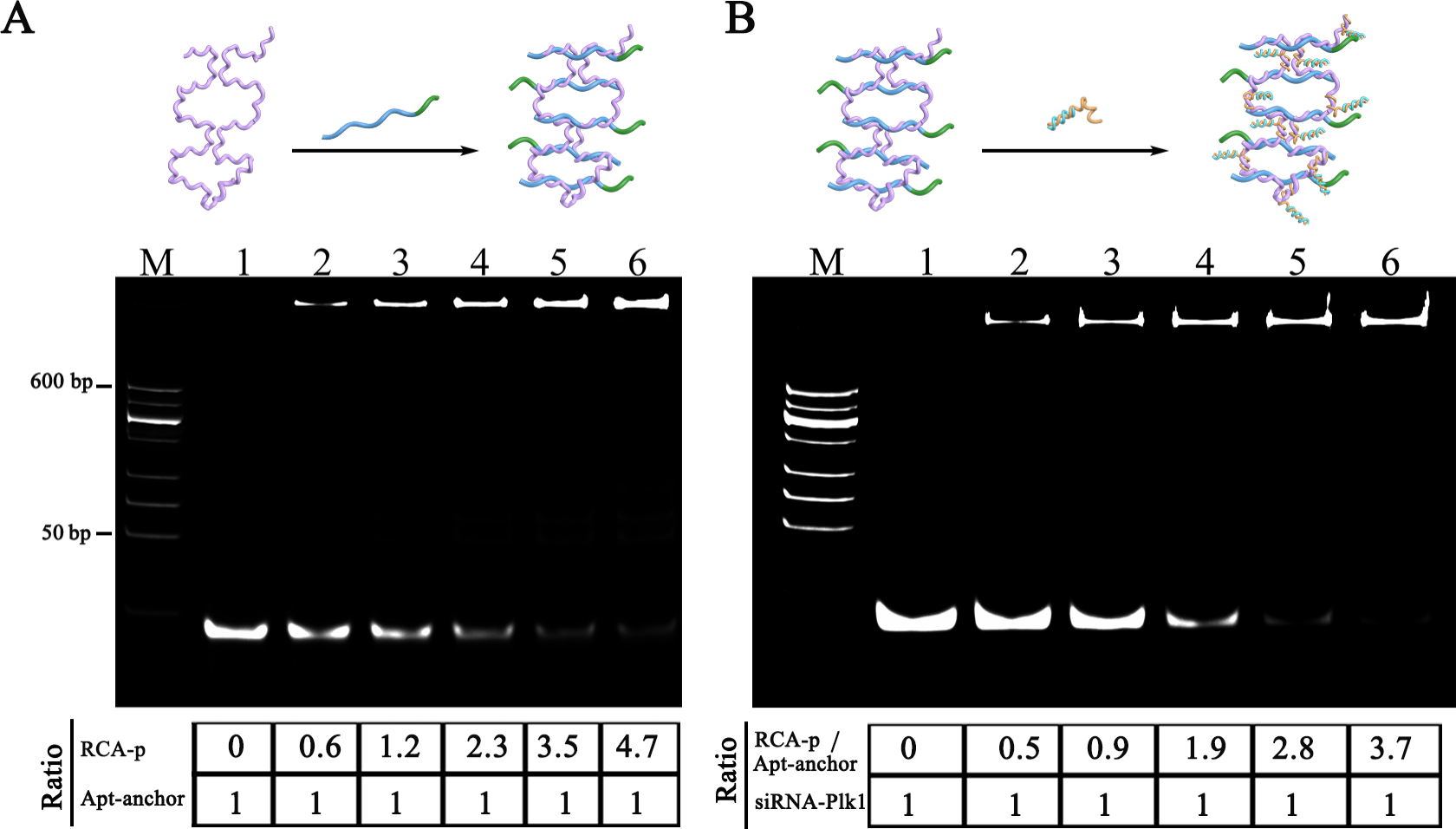


**Figure S2**. **Optimization of the ratio of DNA components.** **(A)** 15% nPAGE analysis of DNA complex assembled by hybridizing Apt-anchor to RCA-p at different molar ratios. When changing RCA-p concentration, the concentration (500 nM) of Apt-anchor remained unchanged. **(B)** 15% nPAGE analysis of products obtained by hybridizing D-siRNA-Plk1 (hybrid of D-Plk1-antisense/D-Plk1-sense) to RCA-p/Apt-anchor hybrids at different molar ratios. The concentration of D-siRNA-Plk1 remained constant (500 nM), while the concentration of RCA-p/Apt-anchor hybrid was gradually increased. RCA-p/Apt-anchor concentration was estimated according to the concentration of RCA-p. The schematic diagram of the hybridization between different DNA components is shown in the upper part.

**Experimental procedure**:

To explore the hybridization ratio between RCA-p and Apt-anchor, 1 μL of 10 μM Apt-anchor was mixed with RCA-p (0 μL, 0.6 μL, 1.2 μL, 2.3 μL, 3.5 μL or 4.7 μL, 10 μM), and the total volume was adjusted with PBS to 20 μL. After annealing at 90 ℃ for 5 min, the resulting solution was cooled down to room temperature and ready for gel electrophoretic analysis.

To optimize the molar ratio of RCA-p/Apt-anchor to D-siRNA-Plk1, RCA-p/Apt-anchor complexes were pre-prepared by mixing Apt-anchor (4 μL, 10 μM) and RCA-p (14 μL, 10 μM) (molar ratio of 1:3.5), and the volume was supplemented with PBS to 40 μL. The resulting mixture was annealed at 90 ℃ for 5 min and cooled down to room temperature. Subsequently, D-siRNA-Plk1 (1 μL, 10 μM) was mixed with a given amount RCA-p/Apt-anchor, and the total volume was adjusted with PBS to 20 μL, followed by incubation at room temperature for 4 h. The finally-assembled products were analyzed by 15% PAGE analysis.


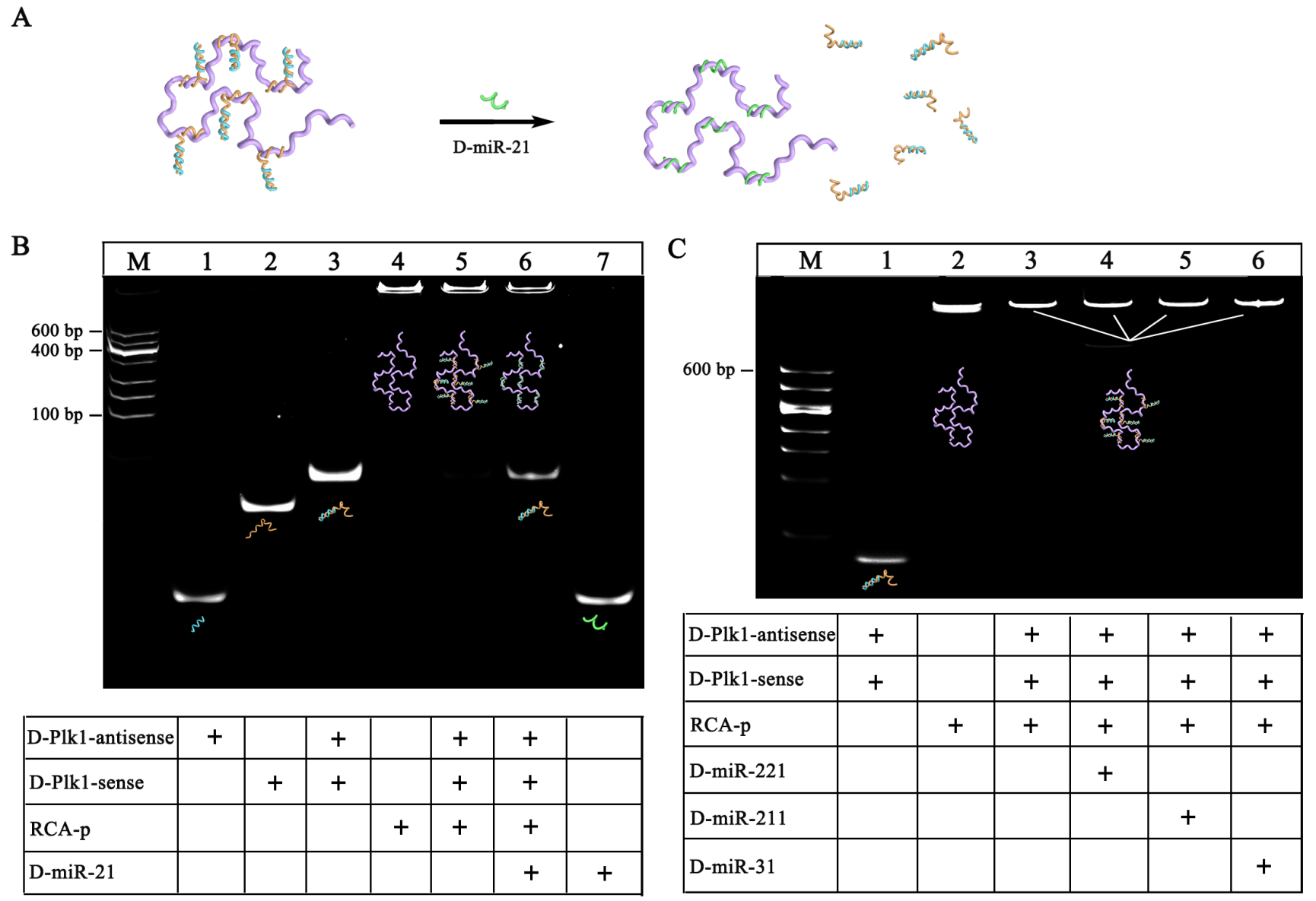


**Figure S3**. **Verification of the release of D-siRNA-Plk1. (A)** The schematic diagram of D-siRNA-Plk1 release from the complex (R-DS) made of RCA-p+D-siRNA-Plk1 mediated by strand displacement reaction. RCA-p indicates RCA products. **(B)** 15% nPAGE analysis to validate the release of D-siRNA-Plk1 from R-DS. Lane 1: D-Plk1-antisense; Lane 2: D-Plk1-sense; Lane 3: D-Plk1-antisense+D-Plk1-sense (D-siRNA-Plk1); Lane 4: RCA-p; Lane 5: RCA-p + (D-Plk1-antisense+D-Plk1-sense) (R-DS); Lane 6: R-DS+D-miR-21; Lane 7: D-miR-21. **(C)** 15% nPAGE analysis to demonstrate the release specificity of D-siRNA-Plk1 towards D-miR-21. Lane 1: D-siRNA-Plk1 consisting of D-Plk1-antisense+D-Plk1-sense; Lane 2: RCA-p; Lane 3: R-DS composed of RCA-p + (D-Plk1-antisense+D-Plk1-sense); Lane 4: R-DS + D-miR-221; Lane 5: R-DS + D-miR-211; Lane 6: R-DS + D-miR-31. The concentration of all DNA sequences was 500 nM. Similar to miR-21, miR-221, miR-211 and miR-31are overexpressed in various malignant tumors.[3, 4]

**Experimental procedure**:

The nPAGE analysis was conducted as described in the section of “***S1.4 Gel electrophoresis***”. The information on the samples is separately shown as follows.

***For lanes 1, 2, 4 and 7 of panel B and Lane 2 of panel C***: A 1-μL aliquot of the sequence of interest (10 μM) was mixed uniformly with 19 μL of PBS (the final volume, 20 μL). Then, the resulting solution was annealed at 90 ℃ for 5 min and cooled to room temperature.

***For Lane 3 of panel B and Lane 1 of panel C***:

D-Plk1-antisense (1 μL, 10 μM) and D-Plk1-sense (1 μL, 10 μM) were added to 18 μL of PBS and mixed thoroughly. After annealing at 90 ℃ for 5 min, the resulting solution was cooled down to room temperature.

***For Lane 5 of panel B and Lane 3 of panel C***: D-Plk1-antisense (1 μL, 10 μM) and D-Plk1-sense (1 μL, 10 μM) were added to 17 μL of PBS and mixed thoroughly, followed by annealing at 90 ℃ for 5 min and cooling down to room temperature. Subsequently, 1 μL of RCA-p was added and mixed. The corresponding product is called R-DS that was stored at room temperature for 4 h.

***For Lane 6 of panel B***: R-DS was prepared as described in Lane 5 except that 16 μL of PBS was involved. Then, 1 μL of D-miR-21 (10 μM) was added and stored for 1 h at room temperature.

***For Lane 4-6 of panel C***: The preparation of samples was the same as ***Lane 6 of panel B*** but D-miR-21 was substituted with D-miR-221, D-miR-211 and D-miR-31, respectively.


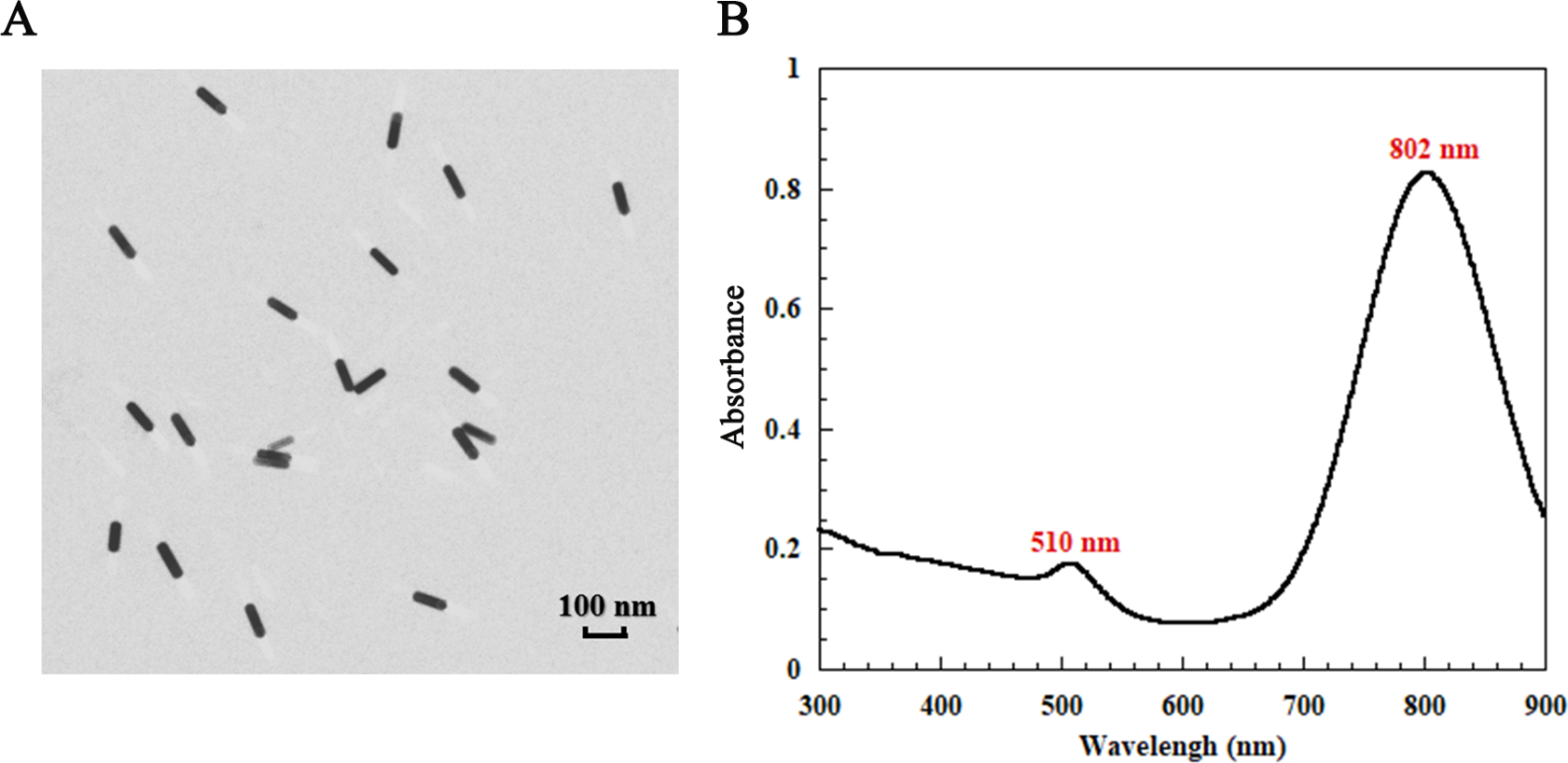


**Figure S4**. Characterization of AuNR. (A) TEM image of AuNR. The scale bar is 100 nm. (B) UV-vis absorption spectrum of AuNR.

**Discussion**

The TEM image of AuNR was performed on a transmission electron microscope (Hitachi 7700, Japan) and the size was estimated by statistically analyzing 20 particles with the help of imageJ software. The resulting information is described as follows: the average particle size of gold nanorods is 79.72 nm ×18.14 nm. UV−vis spectrum of AuNR is consistent with literature results.[5]


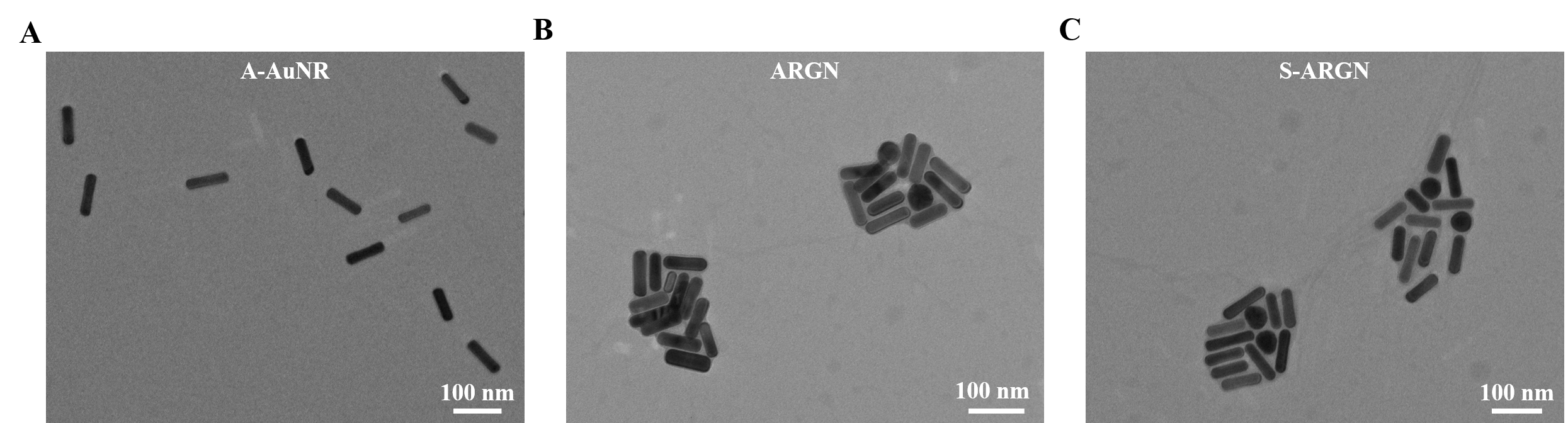


**Figure S5**. **Characterization of S-ARGN and counterparts by transmission electron microscopy (TEM).** TEM images of A-AuNR (A), ARGN (B) and S-ARGN (C). The scale bar is 100 nm.

**Experimental procedure:**

A 10-µL aliquot of analyte sample was dropped on the surface of copper mesh grid coated with thin carbon membrane and incubated for 2 h at room temperature. Then, the copper mesh grid surface was rinsed four times with ddH_2_O (10-µL). Next, 10 μL of 1% phosphotungstate acid was dropped onto the surface of copper mesh grid and incubated for 120 s for negative staining. Afterwards, the solution was removed by contacting the grid side with absorbent paper tissue, and the grid was rinsed four times with ddH_2_O (10-µL). After the resulting copper mesh was dried by a thermostatic metal bath (70 ℃ for 8 h), the TEM imaging was performed on a transmission electron microscope equipment (Hitachi 7700, Japan).


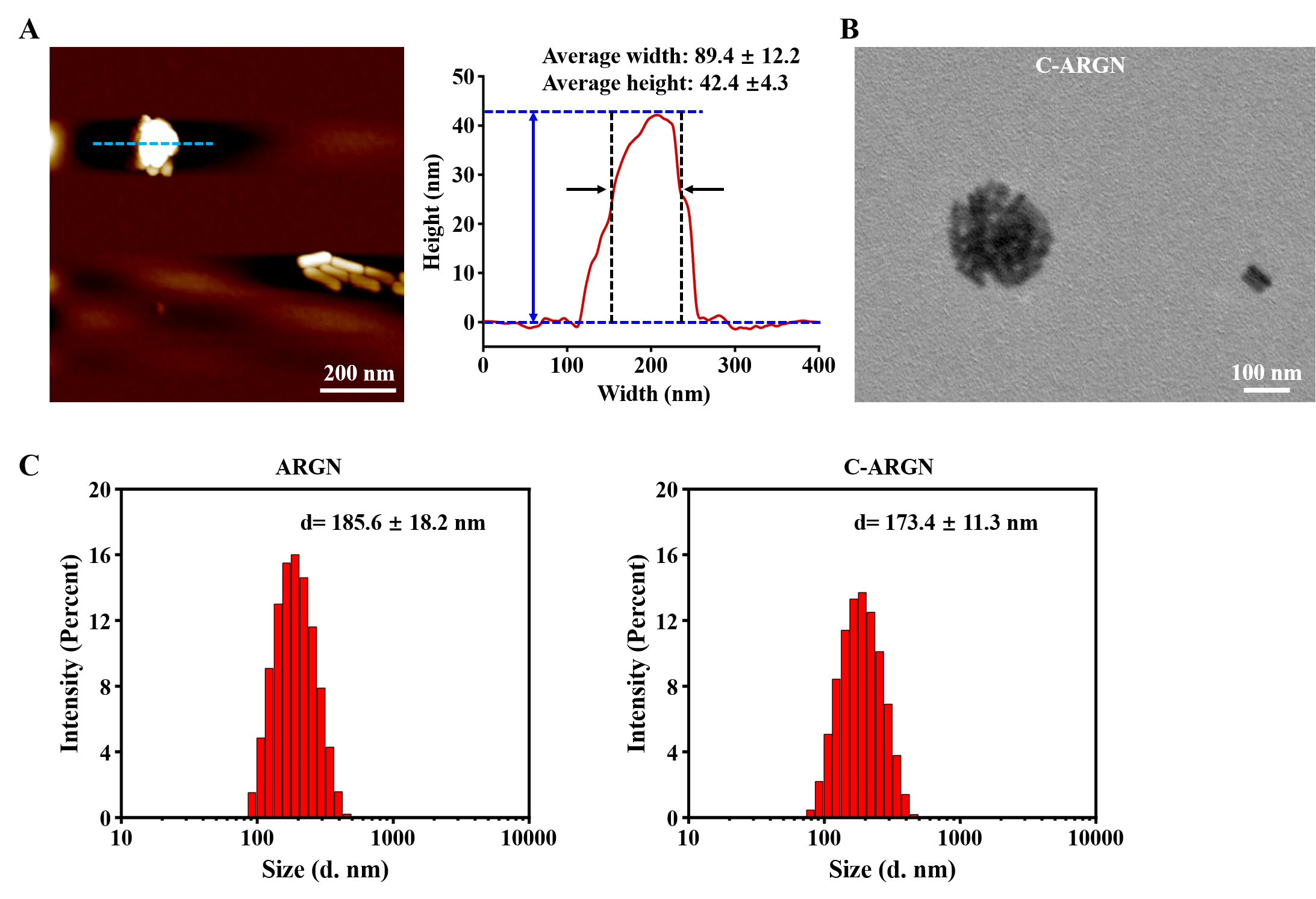


**Figure S6**. **Characterization of C-ARGN, which was essentially ARGN cross-linked with a doubly-thiolated DNA strand crosslinker (D-crosslinker).** (A) AFM images of C-ARGN. The scale is 200 nm. The right part shows the corresponding cross-section profile alone the blue dotted line, accompanied by average width (AW) and average height (AH) (n=5). (B) TEM image of C-ARGN. The scale bar is 100 nm. (C) DLS analysis of ARGN and C-ARGN.

**Experimental procedure:**

*Preparation of C-ARGN:* Firstly, A-AuNR materials were synthesized according to the section of “Preparation of A-AuNR”. After RCA-p (5.19 µL, 10 µM) was mixed with A-AuNR (600 µL, 120 pM), the resulting solution was annealed at 90 ℃for 5 min and slowly cooled down to 25 ℃, generating ARGN. Then, ARGN was centrifuged at 8000 rpm for 5 min. The supernatant was removed and 500 µL of 0.01% tween-20 was used to resuspend the pellet. Next, D-crosslinker (3 µL, 100 µM) and TCEP (1.5 µL, 10 mM) were mixed uniformly and stored at room temperature for 1 h, resulting in Solution C. Subsequently, Solution C was mixed with ARGN, followed by addition of 56.9 µL of sodium citrate (1 M) and incubation for 1 h. The resulting solution was centrifuged, and the pellet was resuspended in 500 µL of PBS. The centrifugation/resuspension step was repeated two times, obtaining C-ARGN that was stored at 4 °C in the dark until used.

*Dynamic light scattering (DLS) analysis:* ARGN and C-ARGN solutions (200 µL) were separately added to 800 µL of ultrapure water and mixed well. DLS measurement was performed on a Nano ZS Zetasizer equipment (Malvern Instruments Ltd., England). Each sample was measured in triplicate.


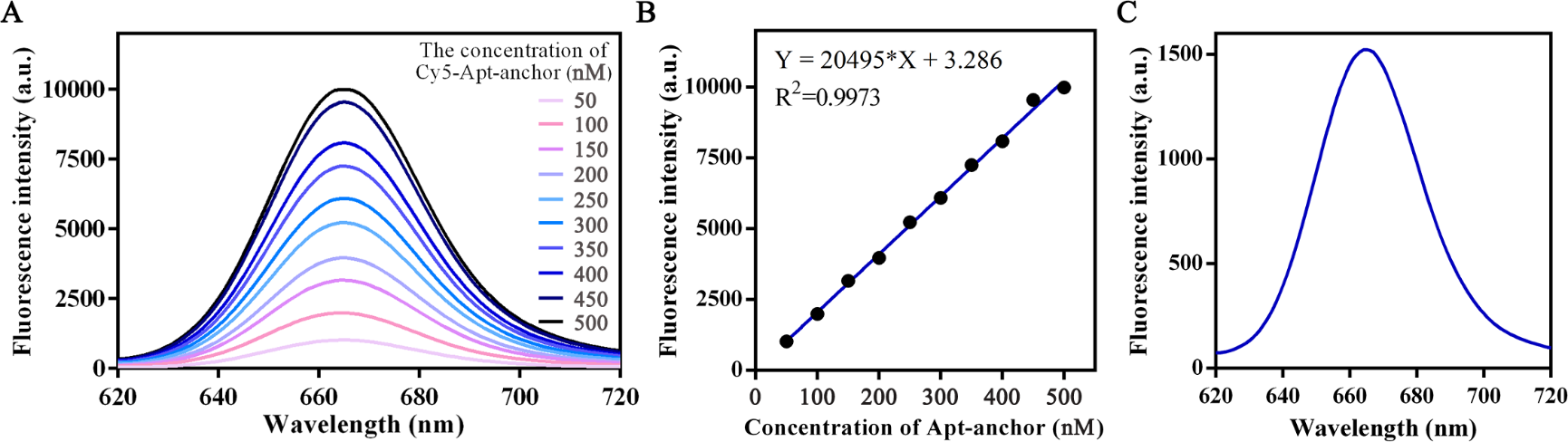


**Figure S7**. **Determination of the number of Apt-anchor on A-AuNR.** (A) Fluorescence spectroscopy of Cy5-Apt-anchor at various concentrations. (B) The linear relationship between the fluorescence intensity at 665 nm and Cy5-Apt-anchor concentration. Inset: the standard linear regression equation. (C) Fluorescence intensity of Cy5-Apt-anchor in the supernatant after incubating A-AuNR with mercaptoethanol and centrifugation.

**Discussion**

To obtain the linear relationship between the fluorescence intensity of Cy5-Apt-anchor at 665 nm and its concentration, different concentrations of Cy5-Apt-anchor solution (200 μL) were prepared, and the fluorescence spectra were collected on a fluorescence spectrometer (Hitachi Ltd, Japan). The experimental results are shown in **Figure S7A**, while **Figure S7B** shows the corresponding standard linear calibration curve that was achieved by comparing the fluorescence intensity at 665 nm with the Cy5-Apt-anchor concentration, accompanied by a standard linear regression equation in the Inset. The fluorescence intensity (1269±179 nM) of Cy5-Apt-anchor released by mercaptoethanol from A-AuNR is shown in **Figure S7C**, which was converted to the molar concentration (C_a_, 61.8±8 nM) by interpolation from standard linear regression equation. The number of Apt-anchor per gold nanorod was calculated to be 206 ± 29 by C_a_ ×200/C_b_×600), where C_b_ (100 pM) indicate the concentration of gold nanorod. The fluorescence measurements (corresponding to the concentration calculated) were independently repeated three times.


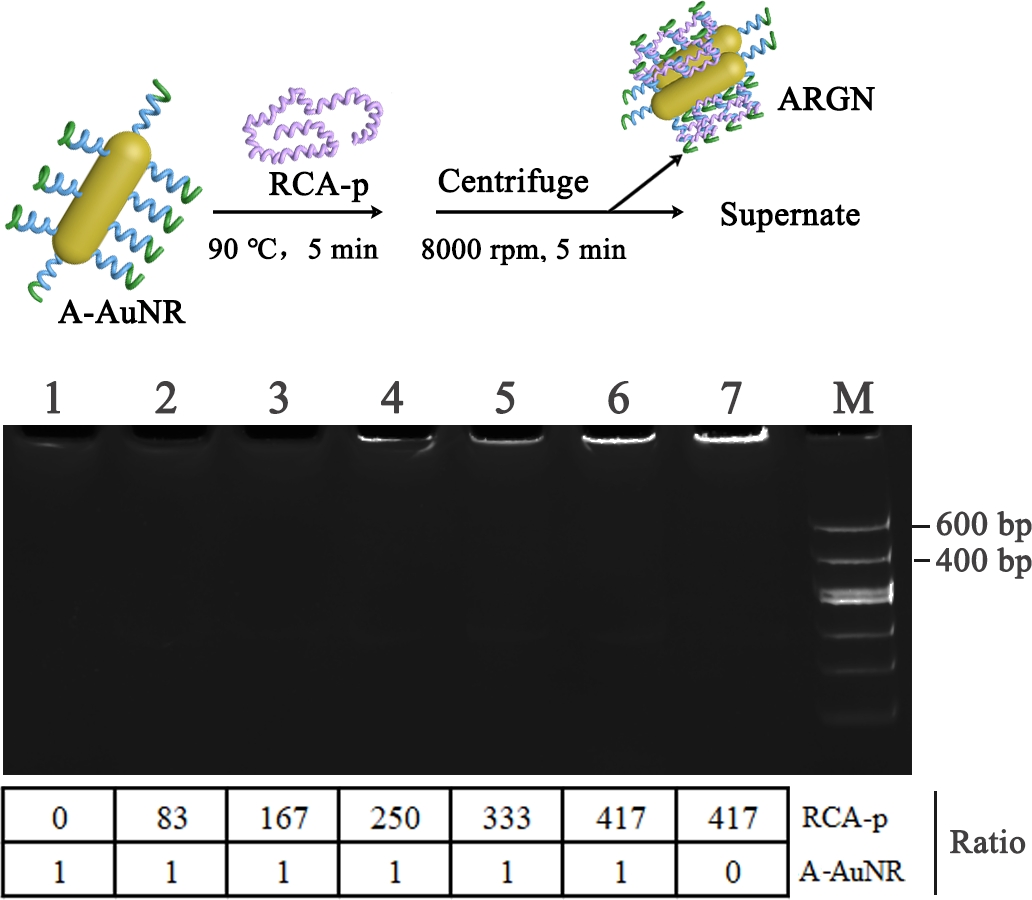


**Figure S8**. **Determination of RCA-p loading capacity**. The upper panel is a schematic diagram of the loading of RCA-p onto A-AuNR, generating the ARGN. The middle panel shows the 15% nPAGE images of supernatants. The low panel shows the molar ratio of AuNR-to-RCA-p corresponding to each lane. The RCA-p concentration estimated from its structural unit concentration is 0 nM, 8.3 nM, 16.7 nM, 25 nM, 33.3 nM or 41.7 nM, while A-AuNR concentration remains unchanged (120 nM).

**Experimental procedure**:

The A-AuNR was pre-prepared as described in the section of ‘Preparation of A-AuNR’ and its concentration is 120 pM. The samples in Lane 1-6 were prepared by mixing the constant amount of A-AuNR (120 pM, 100 μL) with different amounts of RCA-p and the total volumes were adjusted with PBS to 120 μL. The final concentration of RCA-p is 0 nM, 8.3 nM, 16.7 nM, 25 nM, 33.3 nM or 41.7 nM. The resulting solutions were annealed at 90 ℃ for 5 min and then cooled down to room temperature, followed by centrifugation (8000 rpm, 5 min). Afterwards, the supernatants were collected and concentrated to 30 μL at 37 ℃. The nPAGE analysis was conducted as described in the section of “S1.4 Gel electrophoresis”. The sample in Lane 7 was only RCA-p (120 μL, 41.7 nM) as control that was concentrated by the same way before nPAGE analysis.

**Discussion**:

The number of RCA-p loaded onto each AuNR was estimated by electrophoretic measurement. As shown in **Figure S8**, RCA-p was annealed with A-AuNR in different proportions, and the residual amount of RCA-p in the solution was estimated by nPAGE analysis after centrifugation. The band of RCA-p is not observed in Lanes 1-3 but appears in Lane 4 and changes from narrow to wide (Lane 4-7), indicating that RCA-p products were completely loaded at the low ratio of RCA-p to A-AuNR, and the amount of excessive RCA-p gradually increases with the ratio increment. Namely, at the molar ratio of 167:1, RCA-p products are completely loaded, while there are excessive RCA-p products at 250:1. Therefore, the number of RCA-p per A-AuNR is considered to be 167. Taking into account that the theoretical number of the binding sites of each A-AuNR for RCA-p is 206, the loading efficiency of RCA-p is estimated to be 81.1% (167/206×100%), indicating a desirable utilization efficiency of binding sits for RCA-p.


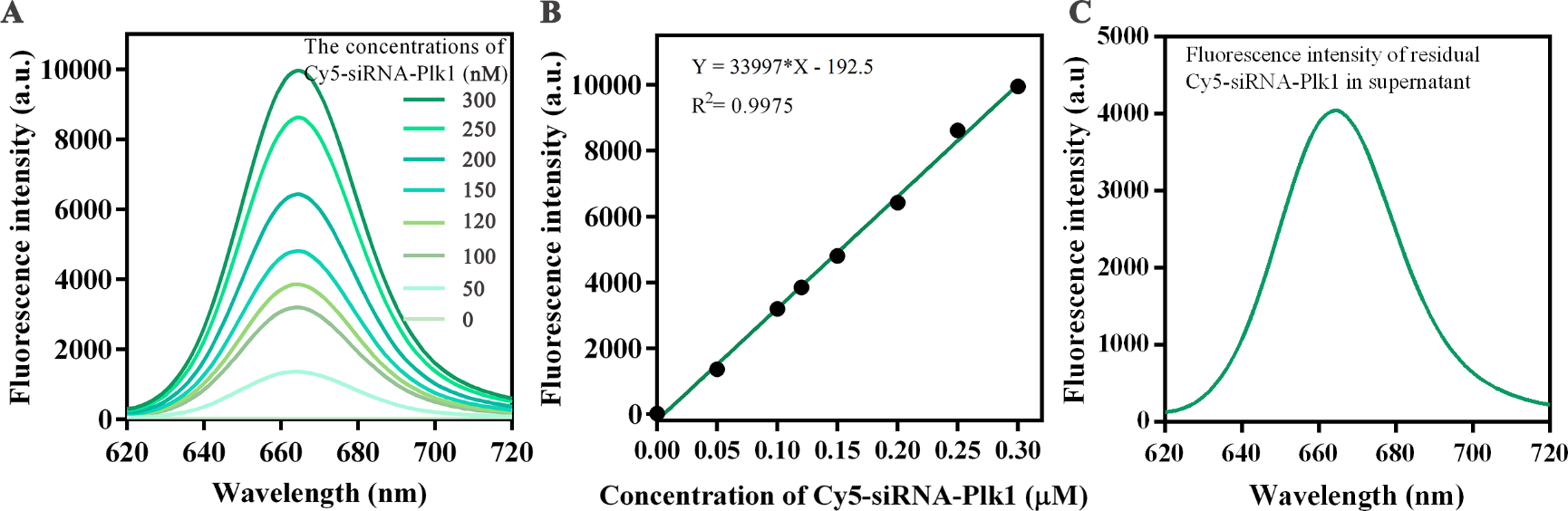


**Figure S9**. **The siRNA loading capability of S-ARGN.** (A) Fluorescence spectra of Cy5-siRNA-Plk1 at different concentrations. (B) The linear relationship between fluorescence intensity at 665 nm and Cy5-siRNA-Plk1 concentration. The inset part is the standard linear regression equation. (C) Fluorescence intensity of the residual Cy5-siRNA-Plk1 in the supernatant after being loaded into ARGN.

**Experimental information and discussion:**

The linear relationship between the fluorescence intensity of Cy5-siRNA-Plk1 at 665 nm and its concentration was explored according to the following procedure: **(i)** Cy5-Plk1-antisense (25 μL, 10 μM) was mixed with Plk1-sense (25 μL, 10 μM), and the volume was adjusted with PBS to 250 μL. After annealing at 90 ℃ for 5 min and cooling to room temperature, Cy5-modifed siRNA complex, Cy5-siRNA-Plk1 was obtained. **(ii)** To prepare a series of standard solutions of known concentration (0 nM, 50 nM, 100 nM, 120 nM, 150 nM, 200 nM, 250 nM and 300 nM), different volumes of Cy5-siRNA-Plk1 were separately diluted with PBS to 200 μL, followed by the fluorescence spectroscopy measurement on a fluorescence spectrometer (Hitachi Ltd, Japan). The excitation wavelength was set at 585 nm, the voltage was 800 V and the excitation/emission slit widths were 10 nm. A standard linear calibration curve was achieved by comparing the fluorescence intensity at 665 nm with the Cy5-siRNA-Plk1 concentration.

The content of siRNA-Plk1 loaded into S-ARGN was determined by the following method. **(i)** The RCA-p (0.75 μL, 10 μM) was mixed with 300 μL of A-AuNRs (120 pM, C_f_) and annealed for 5 min at 90 ℃, followed by slowly cooling to 25 ℃. After centrifugation at 8000 rpm for 5 min, the supernatant was removed and the pellet was resuspended in 200 μL of PBS. **(ii)** Then, 40 μL of Cy5-siRNA-Plk1 (1 μM) was added, mixed thoroughly and stored for 4 h at room temperature. The final concentration of Cy5-siRNA-Plk1 was defined as C_0_ (166.7 nM). The resulting solution was centrifuged (8000 rpm, 5 min) and the supernatant was collected (adjusting the volume to 240 μL), followed by the fluorescence spectroscopy measurement. The fluorescence intensity (4036± 253) was converted to the molar concentration (C_a_, 124.4 ± 7 nM) of Cy5-siRNA-Plk1 by interpolation from a standard linear calibration curve (**Figure S9B**). The content of siRNA-Plk1 was calculated to be 282 ± 50 per AuNR of S-ARGN by (C_0_-C_a_) ×240/(C_f_×300). The fluorescence measurement (used to estimate the concentration) was repeated three times.


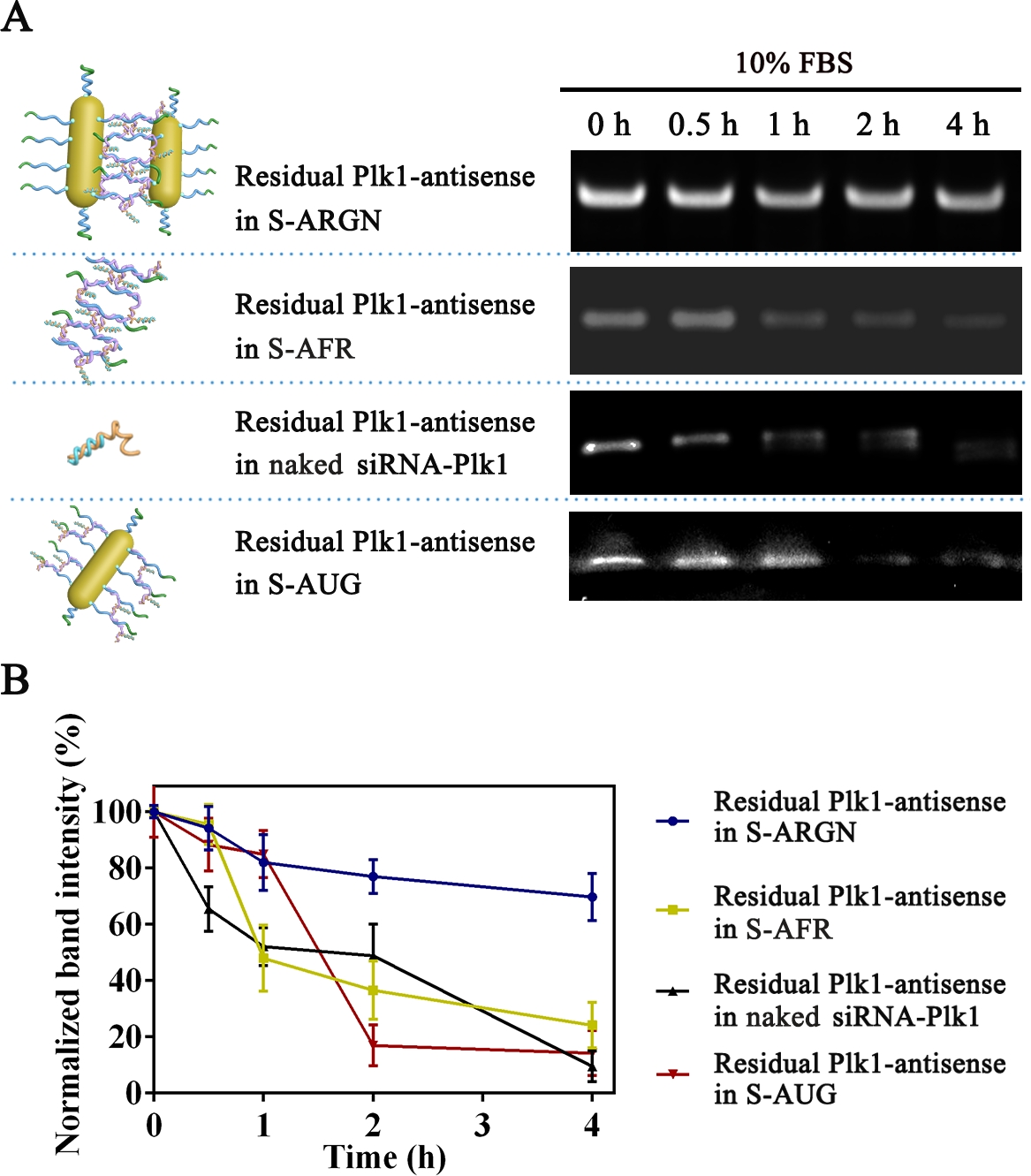


**Figure S10**. **The nuclease degradation-resistance of S-ARGN.** **(A)** The dPAGE analysis (12%) to estimate the amount of residual Plk1-antisense loaded in S-ARGN and its counterparts incubated in 10% FBS for different time periods. **(B)** Quantitative analysis of fluorescence intensity of the band of residual Plk1-antisense in samples presented in panel A with the help of Imagej software. Compared with S-ARGN, S-AFR has no gold nanorods, while RCA-p was substituted with its structural unit of (SUR) in S-AUG.

**Experimental procedure**:

***For S-ARGN***: After S-ARGN (120 μL, 120 pM) was centrifuged (8000 rpm, 5 min), the supernatant was discarded and the precipitate was resuspended in 6 μL of PBS.

***For S-AFR***: Apt-anchor (0.3 μL, 10 μM) and RCA-p (0.24 μL, 10 μM) were added to 1.36 μL of PBS and mixed. The solution was annealed at 90 ℃ for 5 min and cooled to room temperature. Next, 4.1 μL of pre-prepared D-siRNA-Plk1 (1 μM) was added and stored at room temperature for 4 h.

***For siRNA-Plk1***: D-Plk1-antisense (0.4 μL, 10 μM) and D-Plk1-sense (0.4 μL, 10 μM) were added to 5.2 μL of PBS and mixed. The resulting solution was annealed at 90 ℃ for 5 min and then cooled down to room temperature.

***For*** ***S-AUG***: S-AUG was pre-constructed according to the same procedure as S-ARGN but the Structural unit of RCA product (SUR) was used instead of RCA-p. Then, S-AUG (120 μL, 120 pM) was centrifuged (8000 rpm, 5 min). The supernatant was discarded, and the precipitate was resuspended in 6 μL of PBS.

The denatured polyacrylamide gel electrophoresis (nPAGE, 12%) was performed on a gel electrophoresis instrument (Bio-Red, USA). The samples prepared above were incubated with 0.67 mL of fetal bovine serum (FBS) at 37 ℃ for a given time period (0 h, 0.5 h, 1 h, 2 h or 4 h). Then, 6 μL of 2 × loading buffer was added and annealed at 90 ℃ for 5 min. When cooling down to room temperature, the samples were separately loaded into the gel wells and gel electrophoresis was run at 80 V for 90 min in 0.5 ×TBE buffer on ice. Next, the gel was placed in the staining solution consisting of Ultra GelRed (15 μL) and 50 mL of 0.1 M NaCl solution and incubated at room temperature for 15 min, completing the staining process.

**Discussion:**

The endonuclease degradation resistance of siRNA-Plk1 encapsulated in S-ARGN was explored by incubating in 10% fetal bovine serum and comparing with other counterparts. As shown in **Figure S10**, the degradation rate of siRNA-Plk1 loaded in S-ARGN is the slowest, and the residual amount of siRNA-Plk1s remained more than 70% after 4-h incubation in FBS. In contrast, the residual amounts of S-AFR, S-AUG and naked siRNA-Plk1 are about 22%, 15% and 5%, respectively. The experimental results show that S-ARGN can offer the strong protection of siRNA-Plk1 against nuclease degradation.


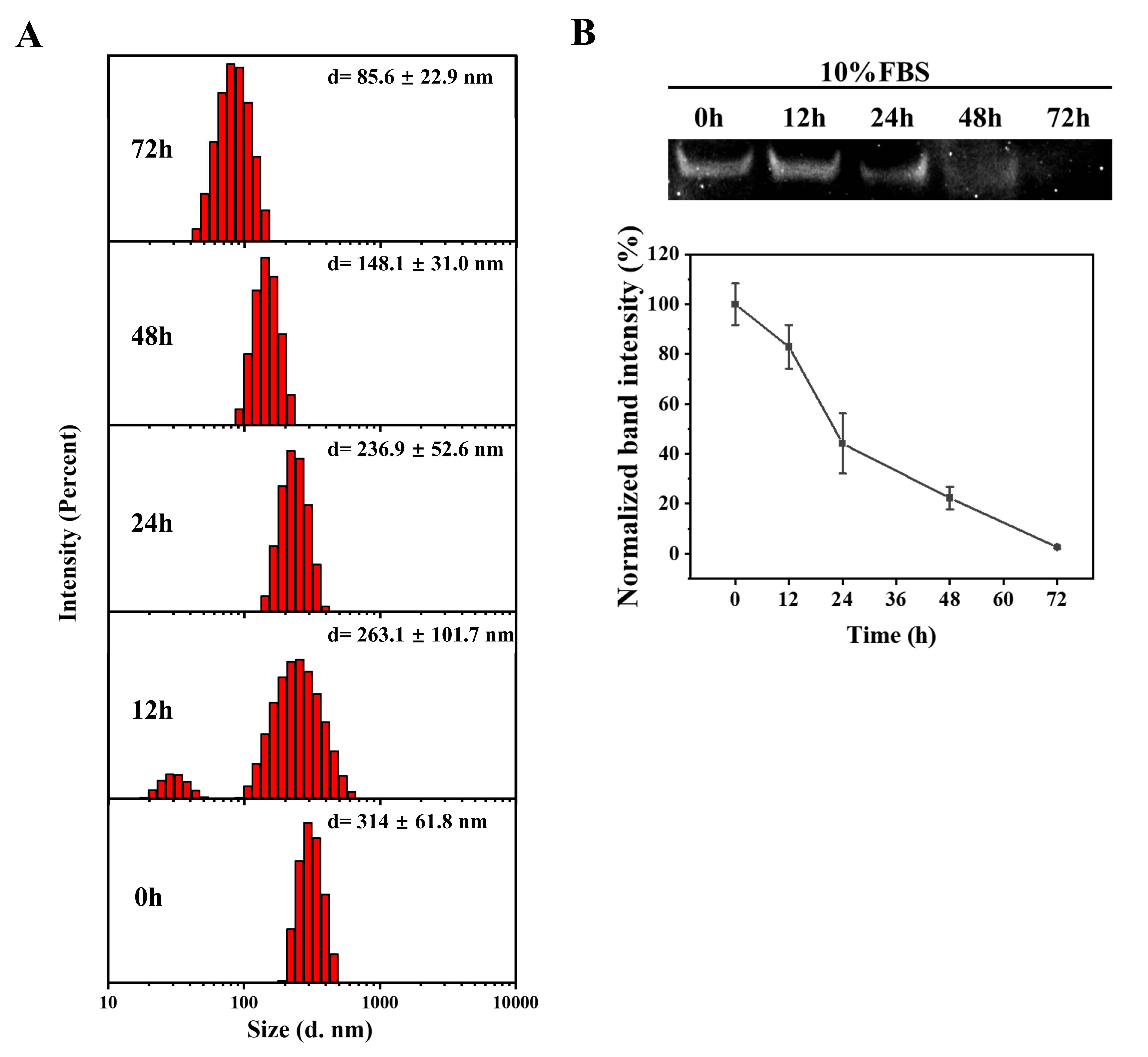


**Figure S11**. **Exploration of the long-term stability of S-ARGN.** **(A)** DLS analysis of S-ARGN samples separately incubated in 10% FBS for 0, 12, 24, 48, and 72 h. **(B)** The dPAGE (12%) analysis to estimate the residual quantity of Plk1 antisense loaded on S-ARGN after incubation in 10% fetal bovine serum (FBS) for 0, 12, 24, 48, and 72 h. Moreover, ImageJ software was utilized to conduct a quantitative analysis of the fluorescence intensity of the residual Plk1 antisense bands in the gel image.


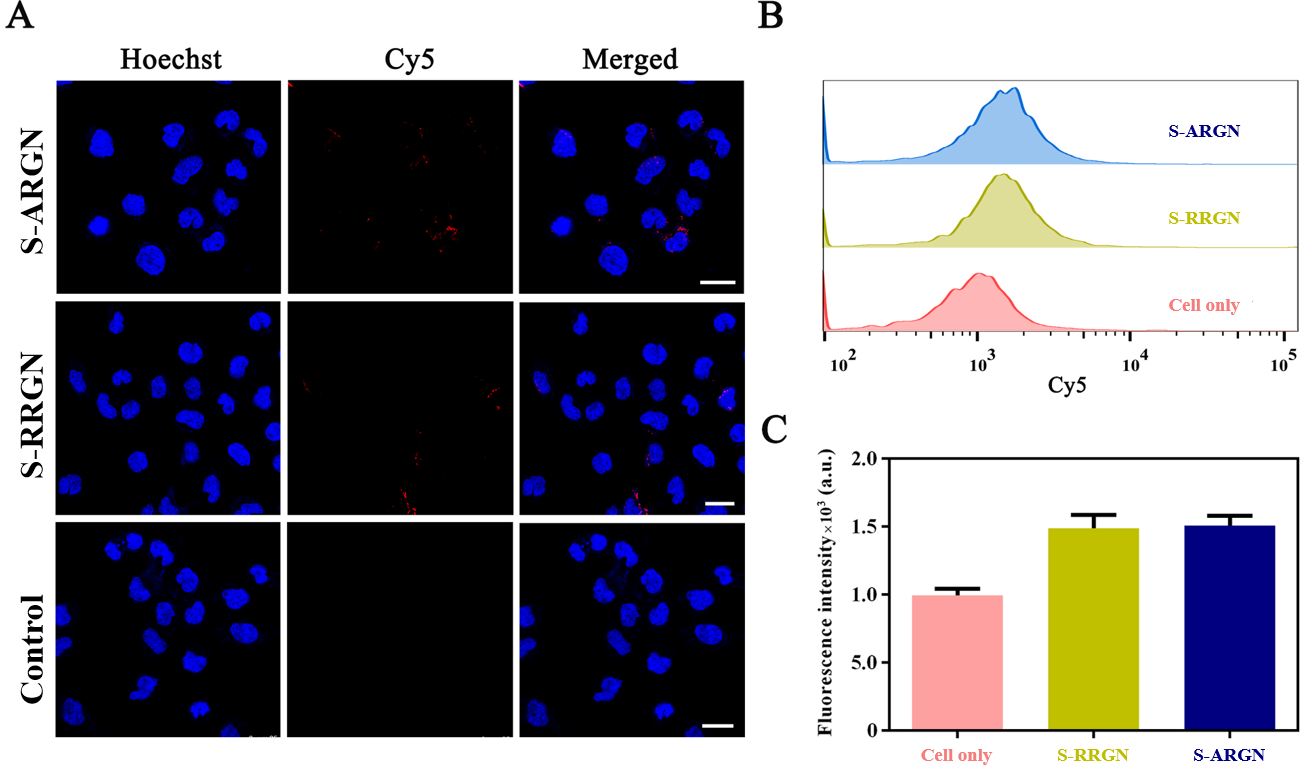


**Figure S12**. **The internalization efficiency of S-ARGN into L02 cells.** **(A)** Confocal fluorescence image of L02 cells treated with S-ARGN (Cy5-siRNA-Plk1 involved during the assembly) for 4 h. The scale is 25 μm. **(B)** Flow cytometry analysis of L02 cells treated with S-ARGN. **(C)** Quantitative analysis of the fluorescence intensity of the samples mentioned in panel B. The concentration of S-ARGN was 48 pM. S-RRGN was a counterpart where aptamer AS1411 was substituted with a random sequence.

**
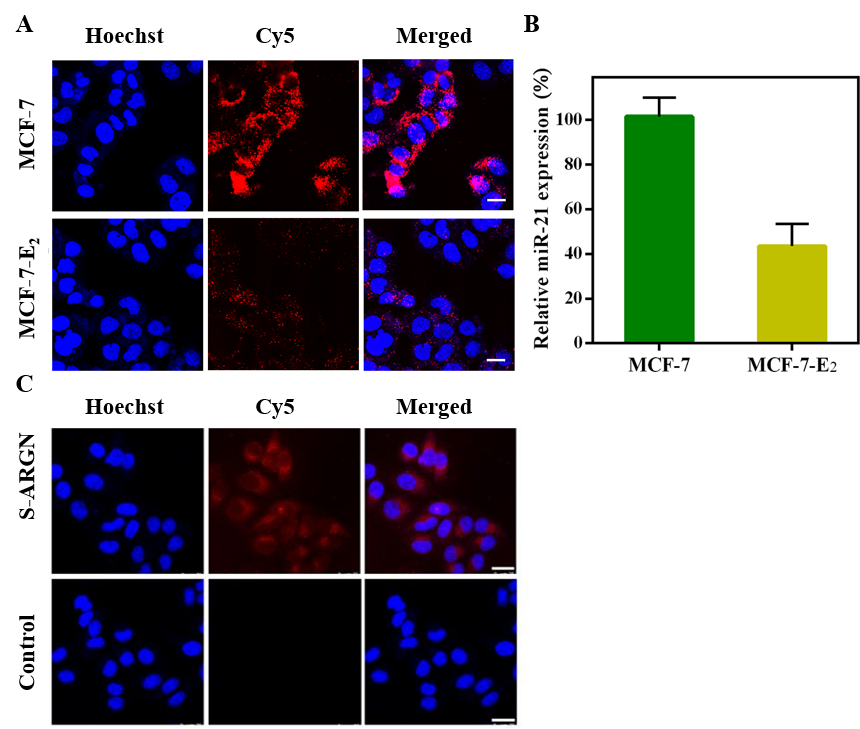
**

**Figure S13**. **The release of siRNA-Plk1 from S-ARGN in response to intracellular miR-21.** **(A)** Confocal fluorescence image of the cells exposed to 4-h treatment with Cy5-labeled S-ARGN where Cy5-Plk1-antisense was hybridized with Plk1-sense to prepare siRNA-Plk1. Compared with MCF-7 cells, MCF-7-E_2_ was pre-treated with estradiol (E_2_) (10 nM) for 24 h to suppress miRNA-21 expression. The scale is 20 μm. **(B)** The relative expression level of miRNA-21 within MCF-7 and MCF-7-E_2_ cells, which was measured by qPCR and estimated by the value of 2^-(∆∆Ct)^. **(C)** The confocal fluorescence image of HeLa cells directly incubated with Cy5-labeled S-ARGN under identical conditions. For Control group, Cy5-labeled S-ARGN was substituted with PBS. The scale is 25 μm.

**Experimental procedure:**

To suppress the expression level of miR-21, β-Estradiol (E_2_) was used to treat MCF-7 cells. Specifically, MCF-7 cells (4.0×10^6^) were incubated with DMEM medium containing 10 nM of E_2_ for 24 h and then washed three times with PBS. The resulting cells were named MCF-7-E_2_. The total miRNA was extracted from MCF-7 or MCF-7-E_2_ (4.0×10^6^) according to the instructions of RNA isolater Total RNA Extraction Reagent. Afterwards, the cDNA was obtained by miRNA 1st Strand cDNA Synthesis Kit (by stem-loop), and qPCR was performed using the miRNA Universal SYBR qPCR Master Mix Kit. Moreover, U6 snRNA was used as the quantitative reference standard. The primers of qPCR are listed in **Table S1**, while the reverse transcription primers are presented below.

Reverse transcription primer for miR-21：GTCGTATCCAGTGCAGGGTCCGAGGTATTCGCACTGGATACGACTCAACA

Reverse transcription primer for U6：

GTCGTATCCAGTGCAGGGTCCGAGGTATTCGCACTGGATACGACAACGCT


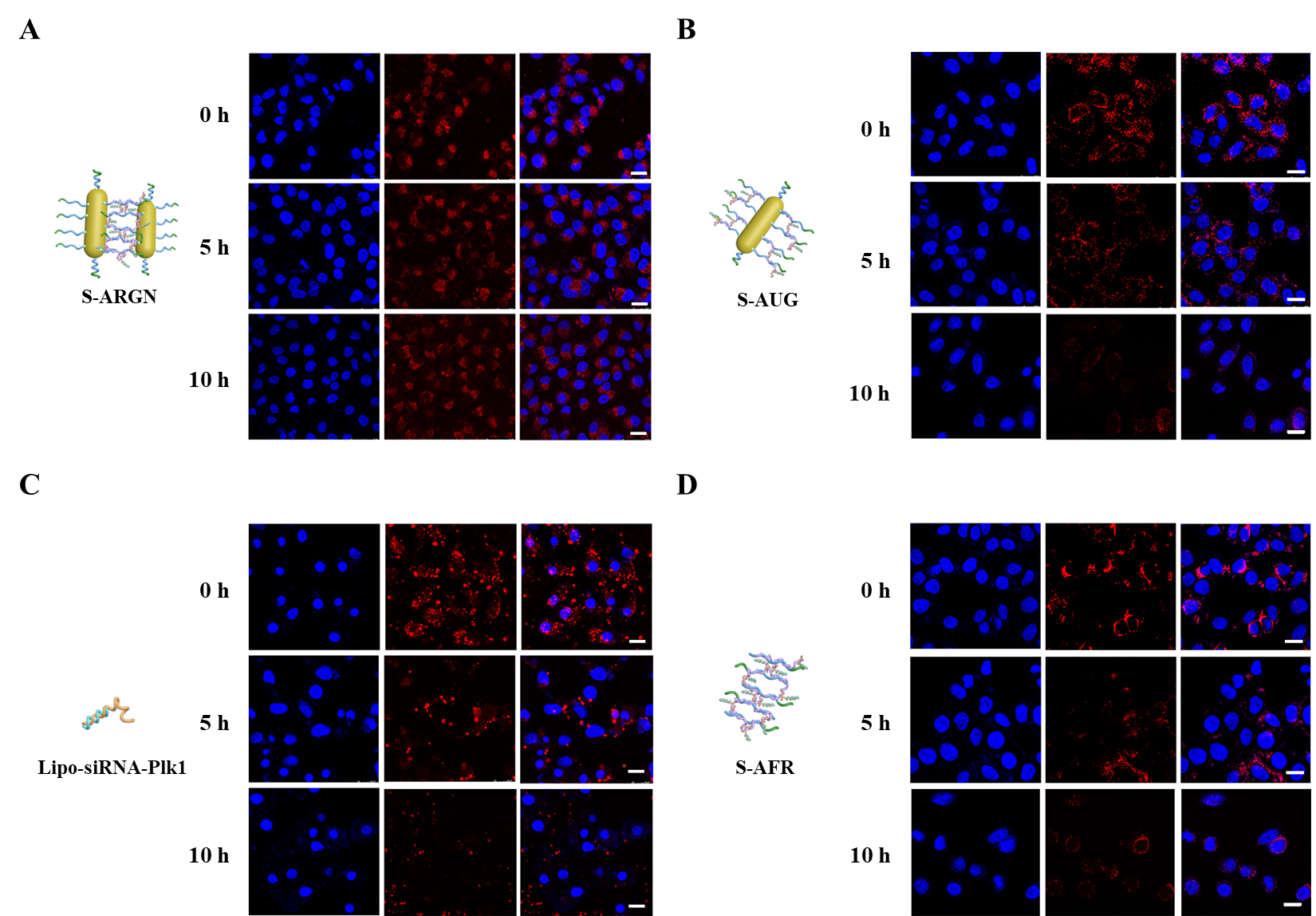


**Figure S14.** **Evaluation of the long-time stability of S-ARGN within living cells.** Confocal fluorescence images of HeLa cells treated as follows. Briefly, after treating with (A) S-ARGN, (B) S-AUG, (C) Lipo-siRNA-Plk1 and (D) S-AFR for 4 h (formulation incubation time, F-time), the used medium was removed. The equivalent concentration of siRNA-Plk1 is 33.3 nM. Then, the resulting cells were incubated in fresh cell culture medium for different time periods (0 h, 5 h and 10 h) (medium incubation time, M-time). The time presented in all panels denotes the medium incubation time rather than the formulation incubation time. The scale is 20 μm.

**Experimental procedure**:

HeLa cells were separately cultured in a 12-well plate at a density of 1.0×10^5^ cells/well. After removal of the medium, the cells were washed with PBS three times and then treated with different nanoformulation as follows.

**For S-ARGN group:** The Cy5-labeled S-ARGN (200 μL, 300 pM) was mixed well with 300 μL of DMED medium and then incubated with cells (one well) for 4 h. After the medium was removed, the resulting cells were cultured in 500 μL of cell medium for different time periods (0 h, 5 h and 10 h).

**For S-AUG group:** The Cy5-labeled S-AUG (200 μL, 300 pM) was well mixed with 300 μL of DMED medium and then incubated with cells (one well) for 4 h. After the medium was removed, the resulting cells were cultured in 500 μL of cell medium for different time periods (0 h, 5 h and 10 h).

**For Lipo-siRNA-Plk1 group**: Transfection experiments were preformed according to the instructions of ExFect2000 Transfection Reagent. Specifically, the cells were washed three times with 500 μL of PBS, followed by addition of 380 μL of medium. ExFect2000 transfection reagent (3 μL) was added into 50 μL of opti-MEM, mixed gently and stored for 5 min. Then, 17 μL of siRNA-Plk1 (1 μM, labeled with Cy5) was added into 50 μL of opti-MEM, mixed gently and stored for 5 min. Subsequently, the two solutions were mixed uniformly and stored for 10 min. The resulting mixture was added into the above-mentioned cultured cells, shaken gently and cultured in an incubator for 4 h. Finally, the culture solution was replaced with fresh culture medium and incubated in an incubator for 0 h, 5 h or 10 h.

**For S-AFR group:** Apt-anchor (1.25 μL, 10 μM) and RCA-p (1 μL, 10 μM) were mixed with 7.75 μL of PBS and annealed at 90 ℃ for 5 min, immediately followed by cooling gradually to room temperature. Subsequently, 17 μL of D-siRNA-Plk1 (1 μM) was added and incubated at room temperature for 4 h, forming the S-AFR. After 173 μL of PBS and 300 μL of cell medium were added and mixed well, the resulting solution was used to culture HeLa cells for 4 h. Next, the used solution was replaced with the fresh culture medium and incubated in an incubator for 0 h, 5 h or 10 h.

For all the groups, the equivalent concentration (33.3 nM) of siRNA-Plk1 was involved, and Cy5-Plk1-antisense was used for the preparation of siRNA-Plk1 to offer a fluorescence signal. Before the fluorescence imaging, the used medium was discarded with a pipette, and the resulting cells were fixed with 4% paraformaldehyde for 15 min and washed three times with 500 µL of PBS. Afterwards, the nuclei were stained with Hoechst 33342 (500 µL, 10 µg/mL) at 37 °C for 15 min and washed with PBS again. Finally, the confocal fluorescence imaging was performed by Leica SP8 laser scanning confocal microscope (Leica, Germany). The excitation wavelengths of 405 nm and 638 nm were used to excite Hoechst and Cy5, respectively.

**Discussion:**

HeLa cells were firstly incubated with fluorescently-labeled siRNA-Plk1-encapsulated formulations for 4 h. After the nanoformulation-contained culture medium was removed, the fresh medium was added and the experimenters started timing. As shown in **Figure S14**, the confocal fluorescence images show that an intense red fluorescence signal is observed at 0 h for S-ARGN, indicating an efficient cellular uptake. With the increment of incubation time to 5 h and 10 h, the intensity of red fluorescence does not change significantly. In contrast, although the three control groups, S-AUG, S-AFR and Lipo-sirNA-Plk1, also show a high red fluorescence signal at 0 h due to the efficient transfection agents or targeting aptamer, a rapid decrease in the fluorescence intensity is detected with the increment of incubation time so that no obvious fluorescence is observed after 10-h incubation, indicating that S-ARGN exhibits the superior effect in terms of intracellular stability to the corresponding counterparts.


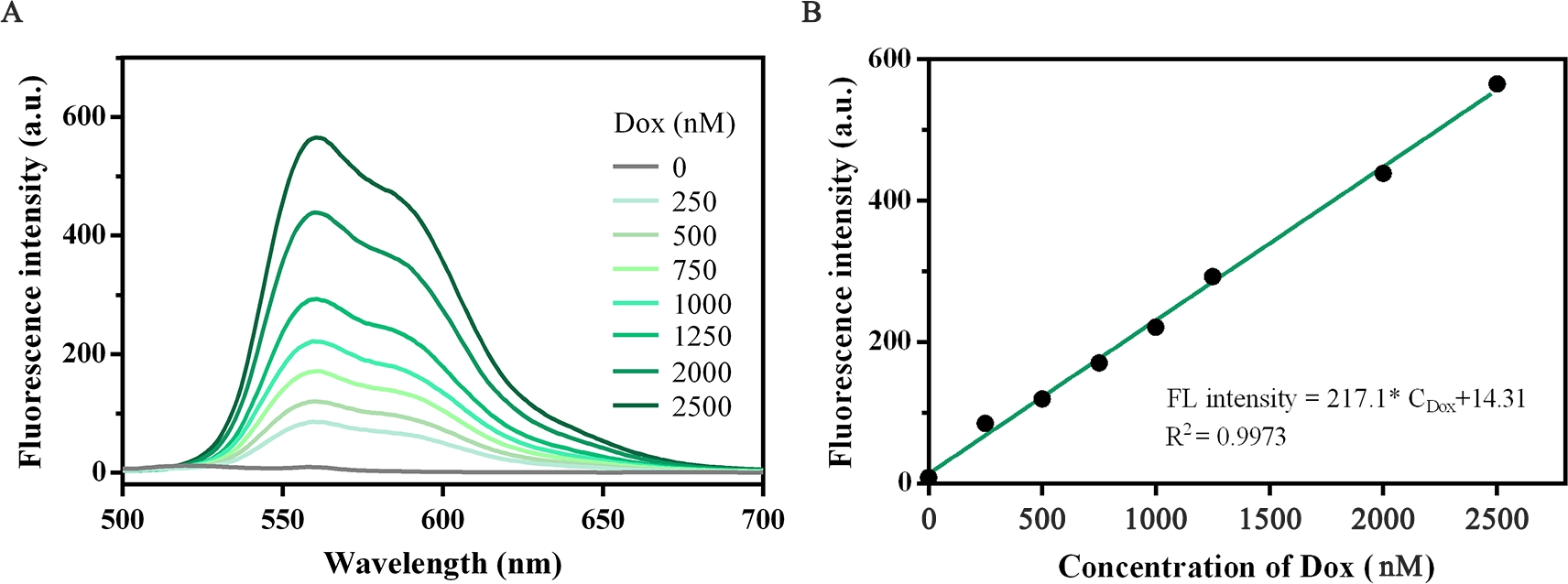


**Figure S15**. **The standard linear calibration curve between Dox fluorescence intensity and its concentration.** (A) Fluorescence spectra of free Dox at various concentrations. (B) The linear relationship between the fluorescence (FL) intensity of free Dox at 560 nm and its concentration. Inset: the standard linear regression equation. C_dox_ represents the Dox concentration.


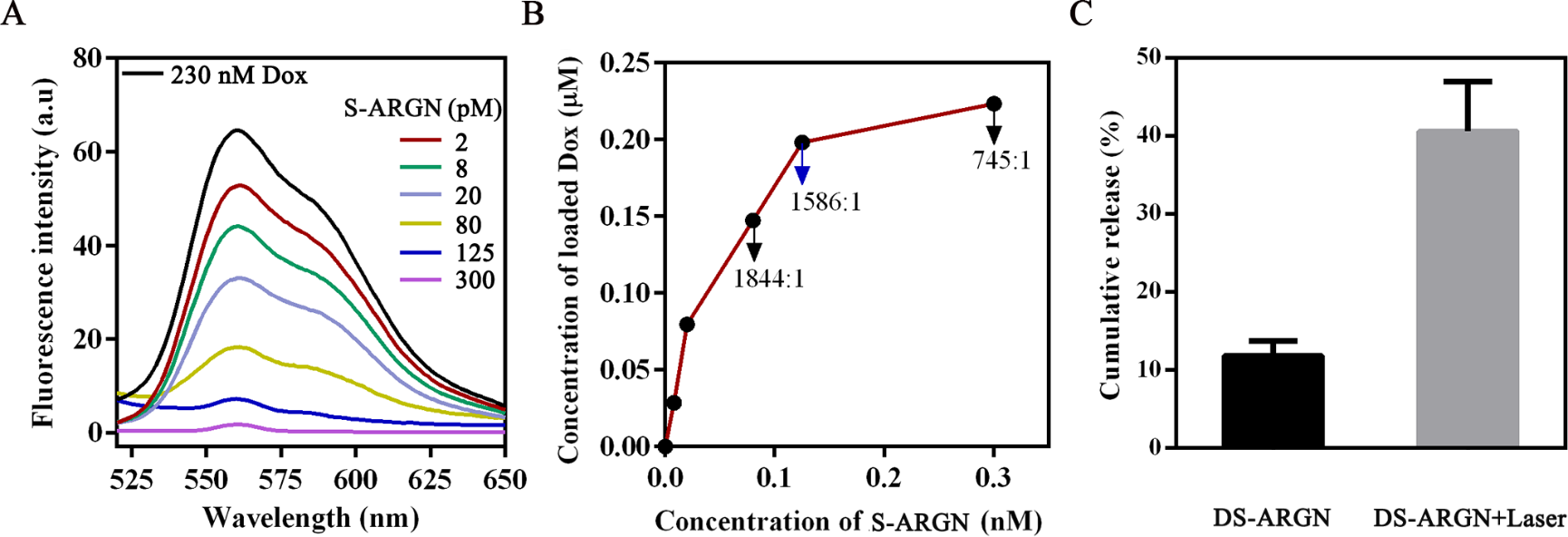


**Figure S16**. **Estimation of Dox loading capacity of S-ARGN and release performance of Dox.** **(A)** Fluorescence spectra of 230 nM Dox in the presence of S-ARGN at different concentrations (2 pM, 8 pM, 20 pM, 80 pM, 125 pM and 300 pM) that was estimated from the initial concentration of AuNRs. **(B)** Dynamic relationship between the concentrations of loaded Dox and S-ARGN. The concentration of Dox loaded into S-ARGN was calculated by subtracting the Dox fluorescence intensity in the supernatant after centrifugation from the initial Dox fluorescence intensity (corresponding concentration, 230 nM) and interpolating from the standard linear calibration curve shown in **Figure S15**. **(C)** Quantitative estimation of the Dox released from DS-ARGN exposed or not exposed to 808 nm laser (0.5W/cm^2^) irradiation. The working solution is PBS.

**Discussion**:

As shown in **Figure S16A**, as the concentration of S-ARGN increases, the fluorescence intensity of Dox gradually decreases, indicating the efficient loading of Dox into the nanoformulation. This is because Dox fluorescence quenching occurs when inserting into the G-C base pairs.[6, 7] As described in **Figure S16B**, the number of Dox per S-ARGN is 1586, which was estimated from the difference in the fluorescence intensity before and after centrifugation according to the standard linear regression equation (**Figure S15**). Moreover, the corresponding loading efficiency of Dox is 86.2%. **Figure S16C** demonstrates that the laser irradiation efficiently promotes the Dox release. Presumably, the temperature increase upon laser irradiation makes the Watson−Crick base-pairing interaction in the double-stranded DNA fragments become fragile and thereby the loaded Dox is easily released.


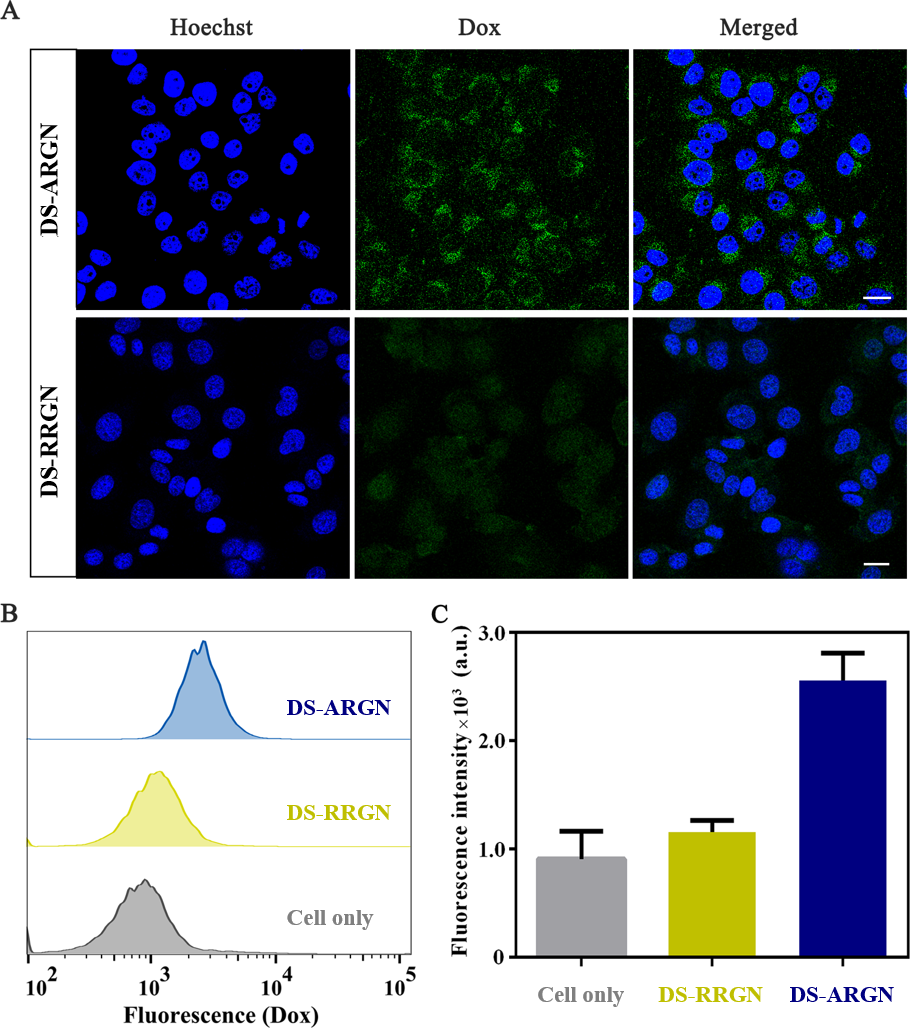


**Figure S17**. **Evaluation of aptamer-dependent tumor cell-targeted Dox delivery of S-ARGN.** (A) Confocal fluorescence imaging of MCF-7 cells incubated separately with DS-ARGN and DS-RRGN for 4 h. The scale bar is 20 μm. (B) Flow cytometry analysis of MCF-7 cells treated with DS-ARGN and DS-RRGN. (C) Quantitative estimation of the fluorescence intensity by FlowJo software corresponding to samples in panel (B). The equivalent concentration of Dox in the finally-prepared solution was 1 μM.


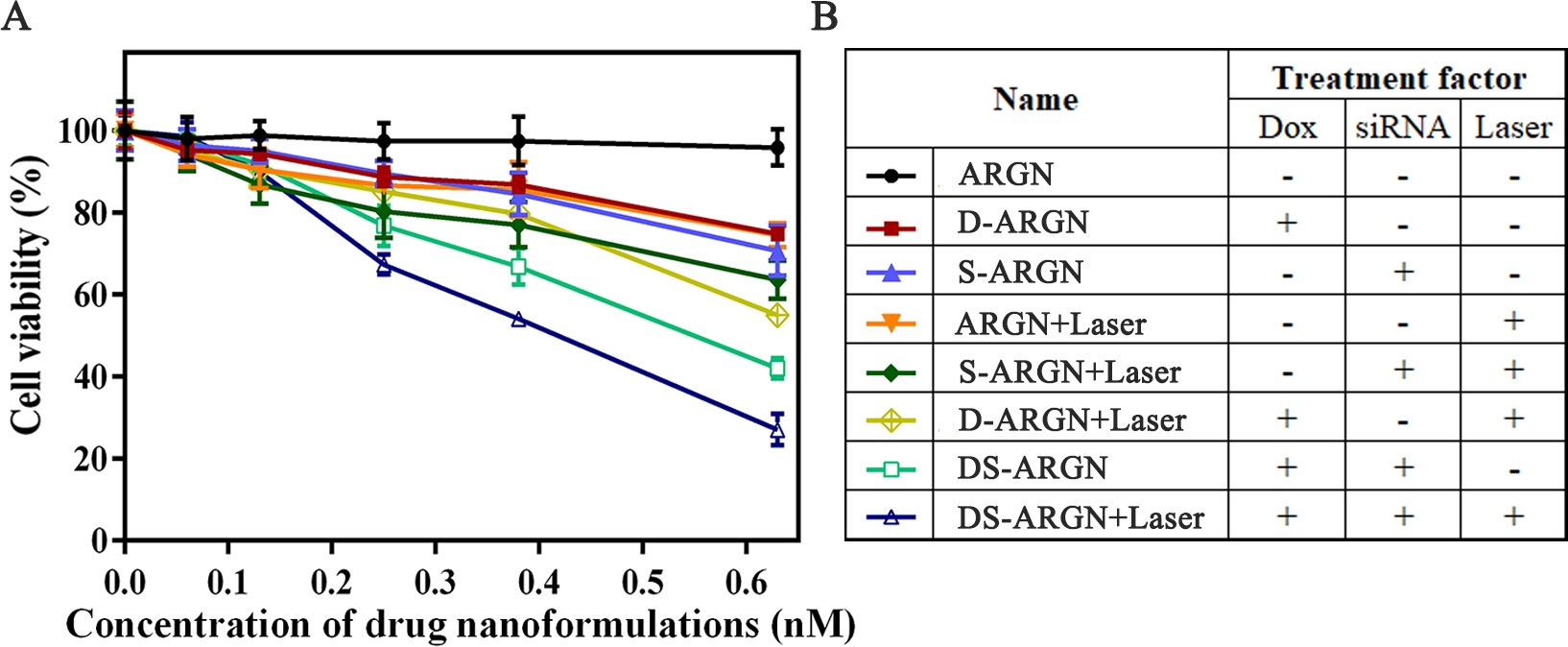


**Figure S18**. **Synergistic effects of multimodal combination cancer therapy.** (A) Cell viability of HeLa cells treated with different nanoformulations at various concentrations for 24 h, which was evaluated by CCK-8 assay. On the horizontal axis indicates the concentration gradient of AuNRs involved in five dose points. For the treatment groups of D-ARGN, D-ARGN +Laser, DS-ARGN and DS-ARGN +Laser, the corresponding equivalent concentrations of Dox are 0 μM, 0.1 μM, 0.2 μM, 0.4 μM, 0.6 μM, and 1.0 μM, respectively. Similarly, for the groups of S-ARGN, S-ARGN+Laser, DS-ARGN and DS-ARGN +Laser, the corresponding equivalent concentrations of siRNA-Plk1 are 0 nM, 17.8 nM, 35.6 nM, 71.1 nM, 106.7 nM and 177.8 nM, respectively. (B) Legends of the nanoformulations involved in panel A. The “siRNA” stands for siRNA-Plk1.

**Figure S19**. **Cytotoxicity of S-ARGN-D combined with 808 nm laser irradiation to cancerous and normal cells.** Three types of cells were treated with different concentrations of S-ARGN-D for 24 h and then subjected to CCK-8 assay following vendor's protocol. Note: in S-ARGN-D, the siRNA-Plk1 was substituted with DNA analogue (D-siRNA-Plk1) and no Dox was involved. From left to right, the concentrations of S-ARGN-D are 0 nM, 0.14 mg/mL, 0.28 mg/mL, 0.56 mg/mL, 0.84 mg/mL and 1.40 mg/mL, in which the corresponding concentrations of DNA products (including RCA-p, D-siRNA-Plk1 and aptamer-anchor) are 0 nM, 40.2 nM, 84.1 nM, 164.4 nM, 248.4 nM and 412.8 nM, respectively.


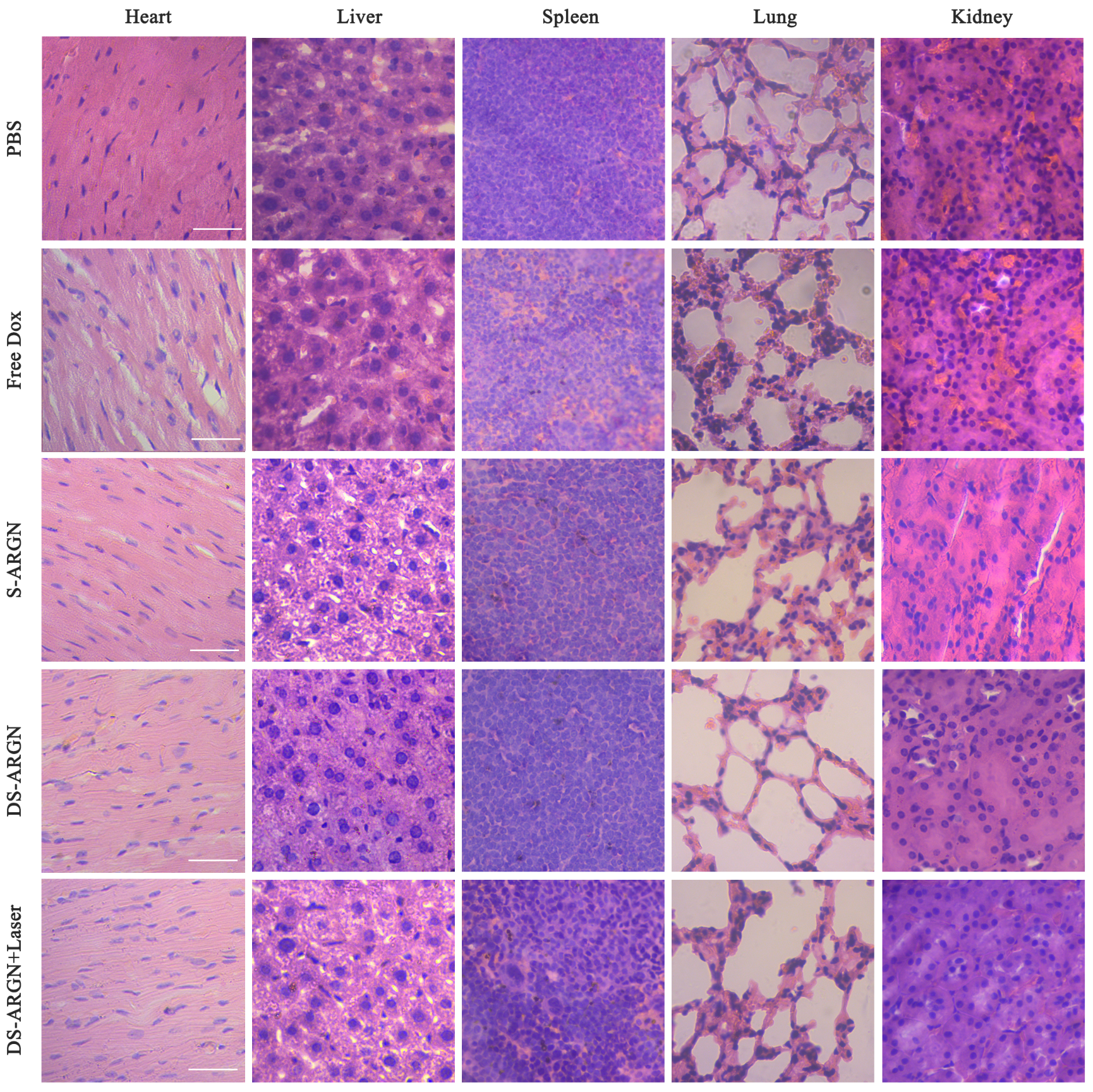


**Figure S20**. **Histological analysis of main organs of HeLa tumor-bearing nude mice by H&E stained sections.** The tissue samples were collected from heart, liver, spleen, lung and kidney of nude mice treated with different drug nanoformulations for 18 d. The scale is 20 μm. The corresponding experimental procedures are described in the section of “Histopathological analysis by hematoxylin and eosin (H&E) staining”.

**Discussion:**

The histological analysis of heart, liver, spleen, lung, and kidney shows that there is no obvious difference in the histological images between DS-ARGN+laser group and saline group, indicating no obvious side effects caused by the damage to normal cells. This is reasonable, besides the accumulation in tumor tissues, the residual DS-ARGNs are easily excreted from the body by liver and kidney as described in Figure 5C. Similar experimental results are obtained for the other two controls, DS-ARGN and S-ARGN. In contrast, free Dox causes the serious damage to normal tissues, especially to heart where obvious myocardial fiber breakage is observed, indicating the unwanted cardiotoxicity.[8, 9] DS-ARGN+ laser exhibits no detectable damage to the five main organs demonstrates that DS-ARGN-based multimodal combination cancer therapy has no systemic toxicity and cardiotoxicity, holding the potential application in targeted cancer therapy.

**S4. Supporting references**

[1] J. Li, B. Zhu, Z. Zhu, Y. Zhang, X. Yao, S. Tu, R. Liu, S. Jia, C.J. Yang. Simple and rapid functionalization of gold nanorods with oligonucleotides using an mPEG-SH/Tween 20-assisted approach [J]. Langmuir, 31(28) (2015) 7869-7876.

[2] S.J. Hurst, A.K. Lytton-Jean, C.A. Mirkin. Maximizing DNA loading on a range of gold nanoparticle sizes [J]. Analytical chemistry, 78(24) (2006) 8313-8318.

[3] T. Tamas, M. Baciut, A. Nutu, S. Bran, G. Armencea, S. Stoia, A. Manea, L. Crisan, H. Opris, F. Onisor. Is miRNA Regulation the Key to Controlling Non-Melanoma Skin Cancer Evolution? [J]. Genes, 12(12) (2021) 1929.

[4] M. Sandesc, A. Dinu, A.F. Rogobete, O.H. Bedreag, D. Sandesc, M. Papurica, L.M. Bratu, S. Negoita, C. Vernic, S.E. Popovici. Circulating microRNAs expressions as genetic biomarkers in pancreatic cancer patients continuous non-invasive monitoring [J]. Clin Lab, 63(10) (2017) 1561-1566.

[5] L. Zheng, X. Hu, H. Wu, L. Mo, S. Xie, J. Li, C. Peng, S. Xu, L. Qiu, W. Tan. In vivo monocyte/macrophage-hitchhiked intratumoral accumulation of nanomedicines for enhanced tumor therapy [J]. Journal of the American Chemical Society, 142(1) (2019) 382-391.

[6] C. Ouyang, S. Zhang, C. Xue, X. Yu, H. Xu, Z. Wang, Y. Lu, Z.-S. Wu. Precision-guided missile-like DNA nanostructure containing warhead and guidance control for aptamer-based targeted drug delivery into cancer cells in vitro and in vivo [J]. Journal of the American Chemical Society, 142(3) (2020) 1265-1277.

[7] S. Zhang, C. Chen, C. Xue, D. Chang, H. Xu, B.J. Salena, Y. Li, Z.S. Wu. Ribbon of DNA lattice on gold nanoparticles for selective drug delivery to cancer cells [J]. Angewandte Chemie International Edition, 59(34) (2020) 14584-14592.

[8] Y. Yang, D. Pan, K. Luo, L. Li, Z. Gu. Biodegradable and amphiphilic block copolymer–doxorubicin conjugate as polymeric nanoscale drug delivery vehicle for breast cancer therapy [J]. Biomaterials, 34(33) (2013) 8430-8443.

[9] Z. Chai, X. Hu, X. Wei, C. Zhan, L. Lu, K. Jiang, B. Su, H. Ruan, D. Ran, R.H. Fang. A facile approach to functionalizing cell membrane-coated nanoparticles with neurotoxin-derived peptide for brain-targeted drug delivery [J]. Journal of Controlled Release, 264(2017) 102-111.
